# One-Pot Synthesis of Bis(arylamino)pentiptycenes by TiCl_4_-DABCO Assisted Reductive Amination of Pentiptycene Quinone

**DOI:** 10.1021/acs.orglett.4c00939

**Published:** 2024-04-24

**Authors:** Zhe-Jie Zhang, Ying-Feng Hsu, Chia-Chien Kao, Jye-Shane Yang

**Affiliations:** Department of Chemistry, National Taiwan University, Taipei, 10617, Taiwan

## Abstract

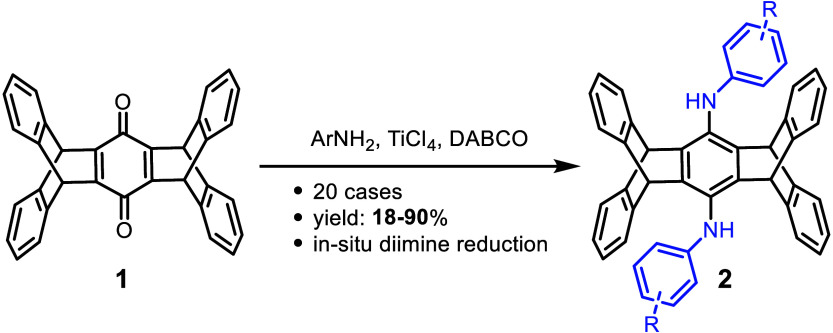

The previously eight-step synthesis of bis(arylamino)pentiptycenes (**2**) from pentiptycene quinone (**1**) can now be achieved in a single step with 18–90% yields through TiCl_4_-DABCO assisted reductive amination with anilines. Both the dual amination of **1** and the *in situ* reduction of quinone diimines are unprecedented. The π system of **2** can be further expanded, including the formation of bis(diarylamino)pentiptycenes. This work also provides mechanistic insights into the challenges encountered in the dual reductive amination of **1** with other amines.

Pentiptycene possesses a rigid H-shaped scaffold, which has found wide utility in supramolecular chemistry and materials chemistry,^1−14^ such as fluorescent sensors,^1−3^ molecular devices,^1,2,4^ host–guest systems,^5,6^ macrocycles,^7^ porous materials,^8−10^ dynamic crystals,^11,12^ and redox-active polymers.^13,14^ The majority of these applications require substitutions on the central phenylene ring of pentiptycene. While the readily prepared pentiptycene quinone (**1**) serves as an ideal precursor for this purpose,^2,15^ some of the derivatization of **1** toward the designated materials suffer from multistep synthesis and low overall yields. For example, bis(4-nitrophenylamino)pentiptycene (**2g**) was a substrate for the synthesis of pentiptycene-containing polyanilines and polyamides,^13,14^ but its synthesis from **1** took eight synthetic steps,^13,16^ resulting in an overall yield of only 15% (Scheme 1a). The lengthy synthetic route arises from the fact that the reaction between **1** and hydroxylamine can only afford the pentiptycene monoxime (**P-ox**) rather than the expected dioxime (**P-diox**) product,^17^ hampering the direct dual reductive amination toward the precursor diaminopentiptycene **3**. Since diphenylamines and triphenylamines are important components in organic electronic materials,^13,14,18^ facile synthetic routes toward pentiptycene-derived diarylamines (e.g., **2g**) and triarylamines are highly desirable.

**Scheme 1:**
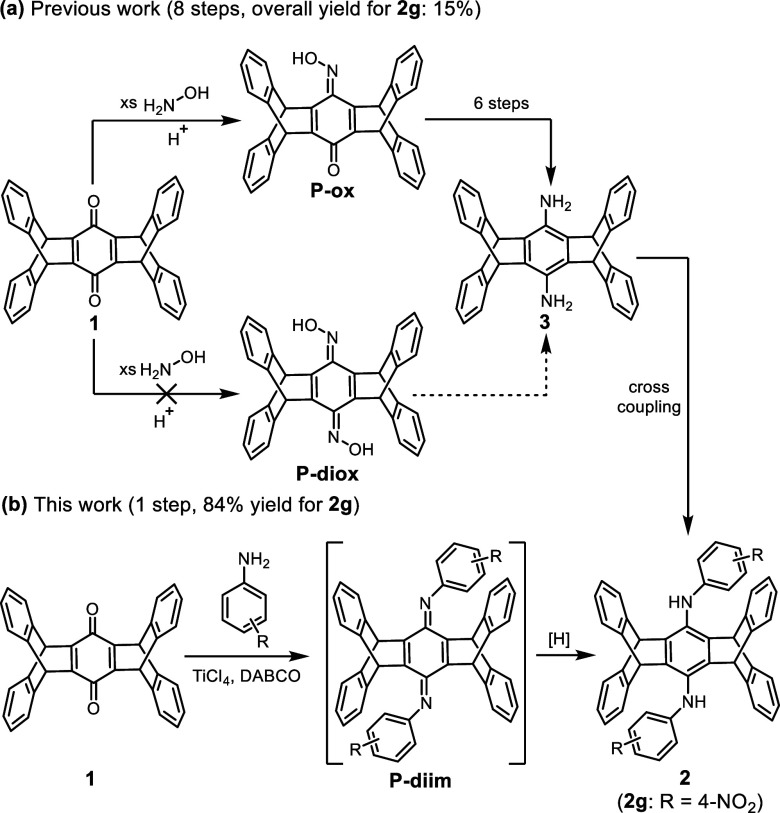
Reductive Amination Reactions of Pentiptycene Quinone (**1**) with (a) Hydroxylamine (Previous Work) and (b) Anilines (This Work)

Here, we report the first case of dual reductive amination of **1**, assisted by TiCl_4_ and DABCO, leading to a series of bis(arylamino)pentiptycenes (**2**) (Scheme 1b). Lewis acid TiCl_4_ has found applications in Baylis–Hillman reaction,^19−21^ Cloke–Wilson rearrangement,^22^ and forming imine macrocycles.^23^ The combination of TiCl_4_ and DABCO is particularly powerful in promoting the condensation of quinones with anilines.^23−30^ In this context, our aim was to investigate whether TiCl_4_ and DABCO can assist in the dual reductive amination of **1** with anilines to afford **2**. Our results reveal an unprecedented *in situ* reduction of the pentiptycene diimines (**P-diim**) under the condensation reaction conditions, enabling a one-pot synthesis of **2**, although the additional step of NaBH_4_ reduction can increase the yield by 2–5%. Subsequent extension of the π-conjugated backbone of **2**, including the formation of triarylamines, can be achieved by S_N_Ar or coupling reactions. This work also provides an explanation for the unfavorable formation of **P-diox**.^17^

We initially employed the conventional two-step protocol,^31^ involving quinone diimine formation followed by reduction with NaBH_4_, for **1** and parent aniline to confirm the feasibility of the reaction. As shown in Table 1, both TiCl_4_ and DABCO are required (entries 1 and 2). The use of 10 equiv of aniline, 6 equiv of TiCl_4_, and 6 equiv of DABCO at 140 °C for 2 days under a N_2_ atmosphere yielded the highest isolated yield of bis(phenylamino)pentiptycene **2a** (91%) among various stoichiometries of aniline, TiCl_4_, and DABCO (entries 3–11).

**Table 1:**
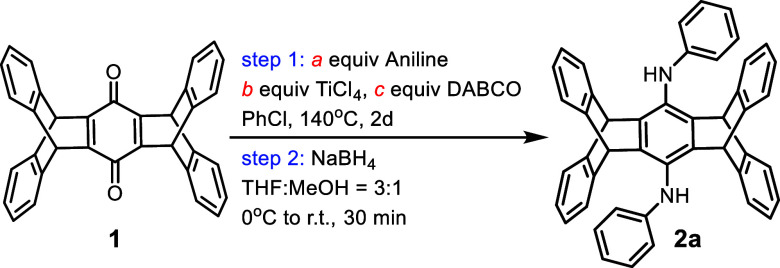
Effects of Stoichiometry of Reagents in the Reductive Amination of Pentiptycene Quinone (**1**) with Aniline^a^

entry	equiv of aniline (*a*)	equiv of TiCl_4_ (*b*)	equiv of DABCO (*c*)	yield (%)^b^
1	10	0	6	0
2	10	6	0	0
3	10	6	6	91
4	8	6	6	84
5	6	6	6	80
6	4	6	6	63
7	2	6	6	28
8	10	5	6	85
9	10	3	6	71
10	10	2	6	60
11	10	6	3	54

a Reaction conditions: (step 1) **1** (1 m mol, 1 equiv, 0.05 M), PhCl (20 mL), N_2_ atmosphere; (step 2) NaBH_4_ (5 equiv), THF (15 mL), MeOH (5 mL), air.

b Isolated yield.

In Table 2, we confirmed that by fixing the stoichiometry of these reagents, TiCl_4_ and DABCO are the optimal Lewis acid and base, respectively, for the preparation of **2a** (entry 1). Substituting TiCl_4_ with other Lewis acids, such as Ti(O^i^Pr)_4_ (entry 2) and ZnCl_2_ (entry 3),^32−35^ resulted in a recovery of starting material **1** with no formation of **2a**. Trimethylamine (TEA) could not replace DABCO in the reaction (entry 4), likely due to the occurrence of α-elimination of amine hydrogen.^36^ In contrast, pyridine gave a comparable yield (entry 5), although its basicity is lower than DABCO. The function of the base was proposed to neutralize the resulting HCl upon the formation of an active titanium/aniline intermediate.^30,36^ DABCO is preferred due to its lower toxicity. Increasing the reaction time did not improve the yield further (entry 6), while decreasing the reaction time lowered the yield (entry 7). At a lower temperature (110 °C), the yield was reduced unless the reaction time was doubled (entries 8 and 9). Interestingly, the reaction in toluene yielded **2a** comparable to chlorobenzene under the same conditions (entries 10 and 11), suggesting that temperature is more critical than solvent in determining the yield (entry 12).

**Table 2:**
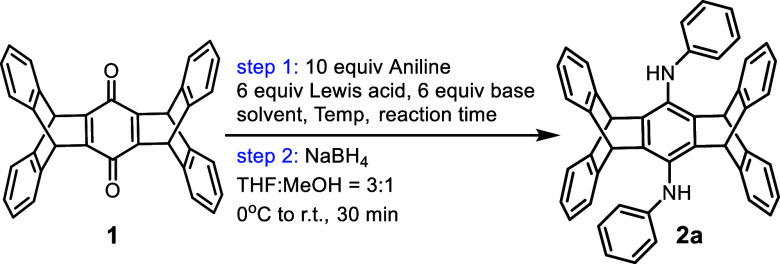
Effects of Reaction Conditions in the Reductive Amination of Pentiptycene Quinone (**1**) with Aniline^a^

entry	Lewis acid	base	solvent	temp (°C)	time (d)	yield (%)
1	TiCl_4_	DABCO	PhCl	140	2	91
2	Ti(O^i^Pr)_4_	DABCO	PhCl	140	2	0
3	ZnCl_2_	DABCO	PhCl	140	2	0
4	TiCl_4_	TEA	PhCl	140	2	0
5	TiCl_4_	Pyridine	PhCl	140	2	90
6	TiCl_4_	DABCO	PhCl	140	4	87
7	TiCl_4_	DABCO	PhCl	140	1	71
8	TiCl_4_	DABCO	PhCl	110	2	78
9	TiCl_4_	DABCO	PhCl	110	4	89
10	TiCl_4_	DABCO	toluene	110	2	73
11	TiCl_4_	DABCO	toluene	110	4	86
12	TiCl_4_	DABCO	PhCl	r.t.	2	0

a Reaction conditions: (step 1) **1** (1 mmol, 1 equiv, 0.05 M), solvent (20 mL, 0.05 M), N_2_ atmosphere. (step 2) NaBH_4_ (5 equiv), THF (15 mL), MeOH (5 mL), air.

b Isolated yield.

However, we later discovered that the vast majority of **P-diim** product was reduced *in situ* under the first-step reaction conditions. For instance, **2a** can be isolated in an 88% yield after the first step, comparable to the two-step protocol’s yield (91%). Notably, this means that **2a** can be prepared in one pot from **1** and aniline without the need for the second step of the NaBH_4_ reduction. This observation is distinctly different from anthraquinone diimine and its analogues, which remain stable under the reaction conditions and necessitate strong reducing agents like hydrazine in the presence of Pd for reduction.^25^ The unprecedented nature of this discovery became apparent when considering that, in the previous TiCl_4_-DABCO assisted condensation of quinones,^24,26−29^ 2 equiv of aniline proved sufficient, but for optimal yield of **2a**, it requires five times as much aniline (10 equiv). In other words, aniline serves not only as a nucleophile for condensation with **1** but also as the reducing agent for the resulting **P-diim**. The oxidation of aniline is indeed supported by the development of a broad absorption band at 400–800 nm,^37^ corresponding to the aniline polymerization (Figure S1). It is worth noting that the **P-diim** cannot be isolated by column chromatography, presumably due to its poor stability (vide infra). Therefore, the crude product obtained from a simple workup of the first step must be directly subjected to NaBH_4_ reduction for the two-step protocol to receive an additional 3% yield of **2a**. The predominant **2a** and minor **P-diim** in the crude of the first step are also supported by the IR spectrum, showing a weak C=N stretching signal at 1655 cm^–1^, with the rest of the spectrum very similar to that of purified **2a** (Figure S2).

Table 3 presents the scope and limitations of the aniline substrate and compares the yield of one-pot vs two-step synthesis. The majority of substituted anilines can produce the corresponding bis(arylamino)pentiptycene (**2b**–**2t**) with moderate to good yields, except for anilines with steric hindrance (**2t**, 18–20%; **2u**, 0%)^32^ or those containing a strong electron-donating substituent (**2m**, 19–23%). It is worth noting that electron-donating substituents have no such negative effect in the benzoquinone and anthraquinone counterparts.^29^ Also note that **2k** is the hydrolyzed product with 4-acetoxyaniline serving as the aniline agent, whereas the pivaloyl group in **2l** can remain intact without undergoing hydrolysis. Similar to the case of **2a**, the yield of one-pot synthesis is comparable (only a 2–5% difference) to that of the two-step protocol in most cases, except for aniline containing a strong electron-withdrawing group such as CN (a 11% difference for **2f**) and NO_2_ (a 15% difference for **2g**). The less efficient *in situ* reduction of **P-diim** in the cases of **2f** and **2g** supports the idea that anilines serve as the reducing agents. On one hand, the strong electron-withdrawing CN and NO_2_ groups are expected to lower the reducing power of 4-cyanoaniline and 4-nitroaniline relative to the parent aniline. On the other hand, these electron-withdrawing substituents can enhance the electron affinity of the resulting **P-diim** such that the *in situ* reduction remains possible. Therefore, the overall effect of electron-withdrawing substituents is to result in a slower *in situ* reduction process. This is supported by both the stronger imine signals in the IR spectrum of the crude samples (Figure S3) and the “recovery” of the comparable yield of one-pot versus two-step synthesis of **2f** (60% vs 61%) and **2g** (84% vs 87%) by increasing the reaction time from 2 to 4 days.

**Table 3:**
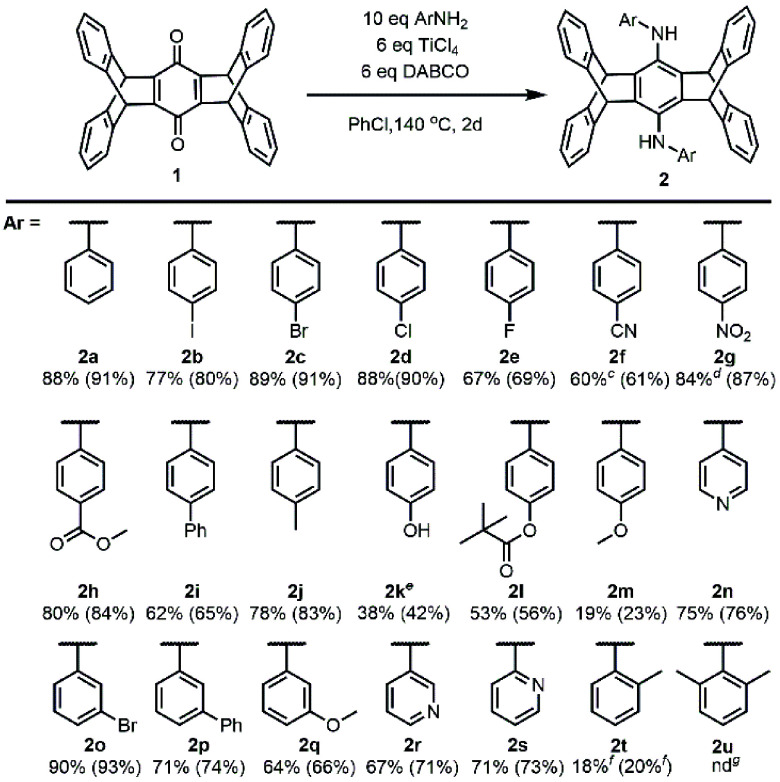
Aniline Substrate Scope in Dual Reductive Amination of Pentiptycene Quinone (**1**)^a^^,^^b^

a Reaction conditions: **1** (1 mmol, 1 equiv, 0.05 M), PhCl (20 mL), N_2_ atmosphere, unless otherwise noted.

b Isolated yield. Values in parentheses refer to the isolated yield from two-step reaction (i.e., the crude products were subjected to NaBH_4_ (5 equiv) in THF (15 mL) and MeOH (5 mL)).

c Reaction time is 4 d (c.f., 50% yield at 2 d).

d Reaction time is 4 d (c.f., 72% yield at 2 d).

e 4-Acetoxyaniline served as the reagent.

f Reaction time: 5 days.

g nd: not detected.

The efficient *in situ* reduction of **P-diim** and the negative electron-donating substituent effect observed for **2m** are reminiscent of the Mills–Nixon effect, a phenomenon involving bond alternation (localization) in strained ring-annelated benzenes, where the annelated bonds tend to have a lower C=C bond character.^38,39^ Since both the annelated bonds in quinoidal **P-diim** (Scheme 1b) and **P-im** (Scheme 2a) are full C=C bonds, the strain in these pentiptycene imines is expected to be larger than that in the benzenoid form. Consequently, there is a higher driving force toward aromatization for **P-diim** and **P-im** relative to those of the benzoquinone and anthraquinone counterparts. While the aromatization of **P-diim** can be achieved by *in situ* reduction, the benzenoid character of the pentiptycene central ring in **P-im** can also be enhanced through resonance, particularly for anilines with electron-donating substituents (e.g., 4-methoxyaniline). As a benzoid form of **P-im** will prevent the second imination toward **P-diim**, this might account for the low yield of **2m**. The same concept can also explain the absence of the **P-diox** formation (Scheme 1a). Once **P-ox** is formed, not only resonance but also tautomerization that forms **P-NO** can occur (Scheme 2b). Supporting this argument is the evidence that the intensity of the C=N vibration peak at 1611 cm^–1^ for **P-ox** is largely diminished when treated with HCl (Figure S4), consistent with acidic conditions favoring the nitroso tautomer.^40^ For comparison, the driving force toward aromatization appears to be much lower in the triptycene quinone counterpart, where only one of the two quinoidal C=C bonds is annelated with a bicyclic ring. This aligns with the fact that the dioxime of triptycene quinone can be isolated.^41^

**Scheme 2:**
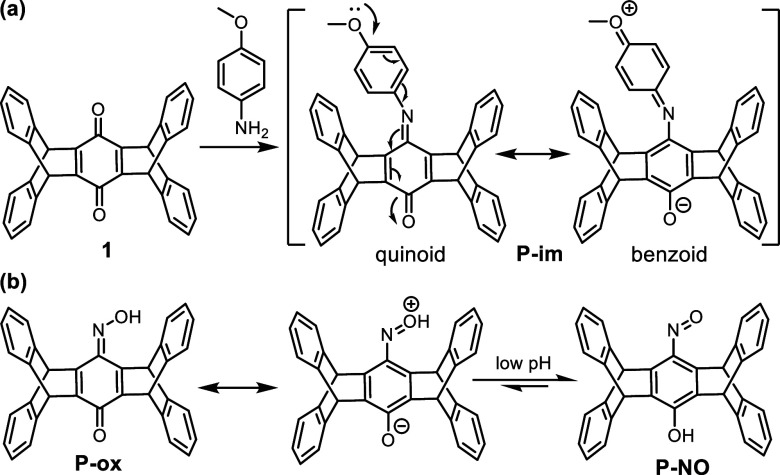
Quinoidal-Benzenoid Resonance Forms of (a) Pentiptycene Mono-Imine and (b) Pentiptycene Mono-Oxime The nitroso tautomer in (b) is also shown.

The feasibility of further extending the π-conjugated system of **2** is demonstrated in Scheme 3. The bromo-substituted **2c** can undergo Heck, Sonogashira, and Suzuki couplings with styrene, phenylacetylene, and phenylboronic acid, respectively. This results in compounds **2v**, **2w**, and **2i** with good yields ranging from 76% to 95%. Furthermore, a microwave-assisted S_N_Ar reaction between **2c** and 1-fluoro-4-nitrobenzene can yield bis(diarylamino)pentiptycene **4**, forming a triarylamine system.

**Scheme 3:**
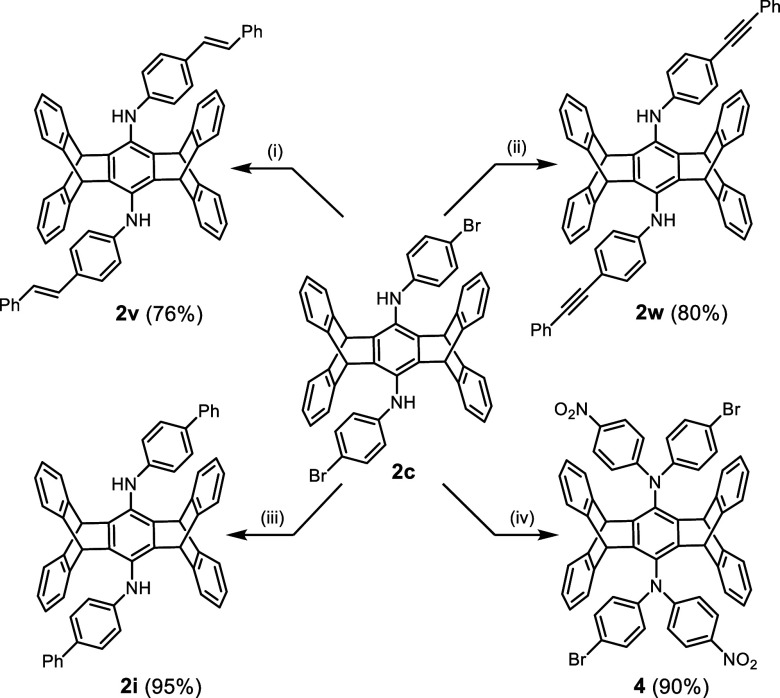
Extension of π-Conjugation of **2c** via Coupling and S_N_Ar Reactions (i) Styrene, Pd(OAc)_2_, Tri-*tert*-butylphosphonium tetrafluoroborate, K_2_CO_3_, DMAc, 130 °C, 20 h, N_2_ atmosphere. (ii) Phenylacetylene, Pd(PPh_3_)_4_, DIPA, Toluene, 90 °C, 20 h, N_2_ atmosphere. (iii) Phenylboronic acid, Pd(PPh_3_)_4_, Cs_2_CO_3_, Dioxane, H_2_O, 100 °C, 20 h, N_2_ atmosphere. (iv) 4-Fluoronitrobenzene, DMSO, microwave 550 W, 100 min.

In summary, we have demonstrated a facile synthesis of bis(arylamino)pentiptycenes (**2**) from pentiptycene quinone **1** with anilines through TiCl_4_-DABCO assisted dual reductive amination. These pentiptycene-containing diarylamines can serve as building blocks for further derivatization toward novel organic electronic materials. Additionally, this work provides new mechanistic insights into pentiptycene quinone chemistry.

## Data Availability

The data underlying this study are available in the published article and its Supporting Information.

## Supporting Information Available

Synthetic details and characterization data and spectra (PDF)
FAIR data, including the primary NMR FID files, for compounds **2a**–**2t**, **2v**, **2w**, and **4** (ZIP)
