# Supplementary material for: One-Pot Synthesis of Bis(arylamino)pentiptycenes by TiCl4-DABCO Assisted Reductive Amination of Pentiptycene Quinone
Source: Org Lett. 2024 Apr 24;26(17):3547–51. doi: 10.1021/acs.orglett.4c00939 (PMC11077485; doi:10.1021/acs.orglett.4c00939)
Supplement: Supplementary file 1 — ol4c00939_si_001.pdf [file ol4c00939_si_001.pdf]

Supporting Information

One-Pot Synthesis of Bis(arylamino)pentiptycenes by $TiCl_4$-DABCO Assisted Reductive Amination of Pentiptycene Quinone

Zhe-Jie Zhang, Ying-Feng Hsu, Chia-Chien Kao, and Jye-Shane Yang*

Department of Chemistry, National Taiwan University, Taipei, 10617, Taiwan

jsyang@ntu.edu.tw

Index

1. General Methods. 4

2. Materials and Synthesis. 4

2.1 General procedures for synthesis of bis(diarylamino)pentiptycenes (2): .4

2.2 Purification method and characterization data of 2a-2t 6

2.3 Procedures for the derivatization of 2c (synthesis of 2v, 2w, 2i and 4)....13

3. Spectra 18

Figure S1. UV-vis spectra of **1**, aniline, **2a**, and crude product of **2a**. 18

Figure S2. FT-IR spectra of crude product of **2a** from one-pot synthesis (upper spectrum) and the isolated **2a** (bottom spectrum). 19

Figure S3. FT-IR spectra of (a) crude product of **2f** from one-pot synthesis (upper spectrum) and the isolated **2f** (bottom spectrum) and (b) crude product of **2g** from one-pot synthesis (upper spectrum) and the isolated **2g** (bottom spectrum). 20

Figure S4. FT-IR spectra of **1** (upper), **P-ox** (middle) and **P-NO** (bottom). 21

Figure S5. ^1H spectrum of compound **2a** ($CDCl_3$, 400 MHz) 22

Figure S6. ^{13}C spectrum of compound **2a** ($CDCl_3$, 126 MHz) 22

Figure S7. ^1H spectrum of compound **2b** (CD_2Cl_2, 400 MHz) 23

Figure S8. ^{13}C spectrum of compound **2b** (CD_2Cl_2, 126 MHz) 23

Figure S9. ^1H spectrum of compound **2c** (CD_2Cl_2, 400 MHz) 24

Figure S10. ^{13}C spectrum of compound **2c** (CD_2Cl_2, 126 MHz) 24

Figure S11. ^1H spectrum of compound **2d** (CD_2Cl_2, 500 MHz) 25

Figure S12. ^{13}C spectrum of compound **2d** (CD_2Cl_2, 126 MHz) 25

Figure S13. ^{1}H spectrum of compound **2e** (CD_2Cl_2, 500 MHz) 26
Figure S14. ^{13}C spectrum of compound **2e** (CD_2Cl_2, 126 MHz) 26
Figure S15. ^{19}F spectrum of compound **2e** (CD_2Cl_2, 471 MHz)........................27
Figure S16. ^{1}H spectrum of compound **2f** (Acetone-d_6, 500 MHz)...................27
Figure S17. ^{13}C spectrum of compound **2f** (Acetone-d_6, 126 MHz)..................28
Figure S18. ^{1}H spectrum of compound **2g** (CD_2Cl_2, 500 MHz) 28
Figure S19. ^{13}C spectrum of compound **2g** (CD_2Cl_2, 126 MHz)........................29
Figure S20. ^{1}H spectrum of compound **2h** (CD_2Cl_2, 500 MHz)........................29
Figure S21. ^{13}C spectrum of compound **2h** (CD_2Cl_2, 126 MHz)........................30
Figure S22. ^{1}H spectrum of compound **2i** (CD_2Cl_2, 400 MHz) 30
Figure S23. ^{13}C spectrum of compound **2i** ($CDCl_3$, 126 MHz) 31
Figure S24. ^{1}H spectrum of compound **2j** (CD_2Cl_2, 400 MHz)..........................31
Figure S25. ^{13}C spectrum of compound **2j** (CD_2Cl_2, 126 MHz)........................32
Figure S26. ^{1}H spectrum of compound **2k** (Acetone-d_6, 400 MHz) 32
Figure S27. ^{13}C spectrum of compound **2k** (Acetone-d_6, 126MHz)..................33
Figure S28. ^{1}H spectrum of compound **2l** (CD_2Cl_2, 400 MHz) 33
Figure S29. ^{13}C spectrum of compound **2l** (CD_2Cl_2, 126 MHz)........................34
Figure S30. ^{1}H spectrum of compound **2m** (CD_2Cl_2, 400 MHz)........................34
Figure S31. ^{13}C spectrum of compound **2m** (CD_2Cl_2, 126 MHz).......................35
Figure S32. ^{1}H spectrum of compound **2n** (Acetone-d_6, 400 MHz)...................35
Figure S33. ^{13}C spectrum of compound **2n** (CD_2Cl_2/ CD_3OD = 5/1, 126 MHz) 36
Figure S34. ^{1}H spectrum of compound **2o** (CD_2Cl_2, 400 MHz) 36
Figure S35. ^{13}C spectrum of compound **2o** (CD_2Cl_2, 126 MHz)........................37
Figure S36. 1H spectrum of compound **2p** (CD_2Cl_2, 400 MHz) 37
Figure S37. ^{13}C spectrum of compound **2p** (CD_2Cl_2, 126 MHz)........................38
Figure S38. ^{1}H spectrum of compound **2q** (CD_2Cl_2, 500 MHz)........................38
Figure S39. ^{13}C spectrum of compound **2q** (CD_2Cl_2, 126 MHz)........................39
Figure S40. ^{1}H spectrum of compound **2r** (DMSO-d_6, 500 MHz) 39
Figure S41. ^{13}C spectrum of compound **2r** (DMSO-d_6, 126 MHz)....................40
Figure S42. ^{1}H spectrum of compound **2s** (Acetone-d_6, 400 MHz) 40

Figure S43. ^{13}C spectrum of compound **2s** (CD_2Cl_2/ CD_3OD = 5/1, 126 MHz) 41

Figure S44. ^{1}H spectrum of compound **2t** (CD_2Cl_2, 400 MHz)........................41

Figure S45. ^{13}C spectrum of compound **2t** (CD_2Cl_2, 126 MHz)........................42

Figure S46. ^{1}H spectrum of compound **2v** (CD_2Cl_2, 500 MHz)........................42

Figure S47. ^{13}C spectrum of compound **2v** (CD_2Cl_2, 126 MHz)........................43

Figure S48. ^{1}H spectrum of compound **2w** (DMSO-d_6, 400 MHz)....................43

Figure S49. ^{13}C spectrum of compound **2w** ($CDCl_3$, 126 MHz) 44

Figure S50. ^{1}H spectrum of compound **4** (DMSO-d_6, 500 MHz).......................44

Figure S51. ^{13}C spectrum of compound **4** (DMSO-d_6, 126 MHz)......................45

4. References..46

1. General Methods.

The **^{1}H-NMR** spectra, **^{13}C-NMR** spectra and **^{19}F-NMR** spectra were recorded by a Bruker AVIII-400 MHz or a Bruker AVIII-500 MHz spectrometer. The chemical shifts (δ) were reported in parts per million (ppm) relative to the solvent used, including $CDCl_3$ (^{1}H: δ = 7.26, ^{13}C: δ = 77.00), CD_2Cl_2 (^{1}H: δ = 5.32, ^{13}C: δ = 54.00), DMSO-d_6 (^{1}H: δ = 2.54, ^{13}C: δ = 39.50), acetone-d_6 (^{1}H: δ = 2.05, ^{13}C: δ = 30.60 and 205.87), and CD_2Cl_2-CD_3OD cosolvent (CD_2Cl_2/CD_3OD =5/1: ^{13}C: δ = 54.00 for the peak of CD_2Cl_2). **Fourier transform infrared (FT-IR)** spectra were recorded on a Thermo Scientific Nicolet iS5 FT-IR spectrometer by using drop-casting method on a KBr pellet. **UV-visible spectra** were recorded using a Cary 300 double-beam spectrophotometer. **High-resolution mass** data were collected by using matrix-assisted laser desorption/ionization time-of-light (MALDI-TOF) with a Bruker Autoflex Speed spectrometer or by electrospray ionization (ESI) with a Bruker micro TOF-QII spectrometer.

2. Materials and Synthesis.

All commercial reagents, catalysts, solvents were used as received. Column chromatography was carried out on silica gel (Geduran® SI 60). The pentiptycene quinone (**1**) starting material was synthesized according to the previous report.[S1] The microwave reaction for the synthesis of **4** was performed by a MLS Milestone MicroMED T/T Mega Rapid Microwave. Compound **2a**,[S2] **2g**,[S3] and **2m**[S4] are known compounds.

2.1 General procedures for synthesis of bis(diarylamino)pentiptycenes (2):

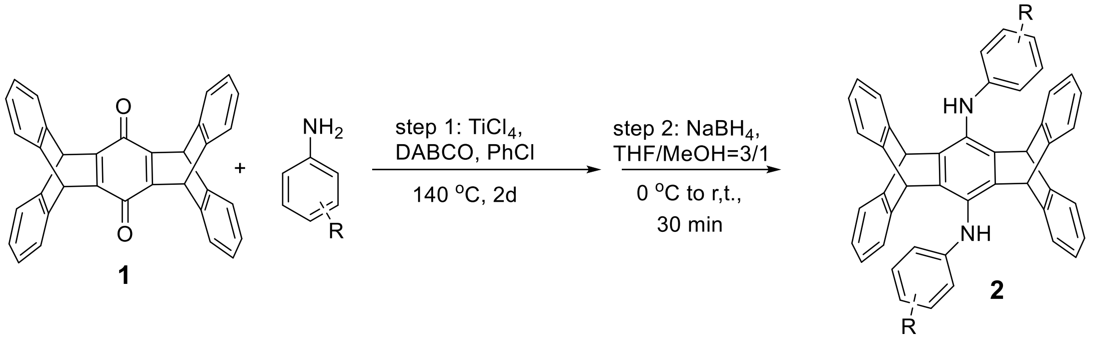

The procedures are illustrated by the synthesis of **2a**, where pentiptycene quinone **1** (460 mg, 1 mmol, 1 equiv) is the limiting reagent. The same procedures can be applied to the synthesis of **2b**-**2t** by changing the anilines.

For one-pot synthesis (step 1 only): To a two-neck bottle equipped with a stirring bar containing the pentiptycene quinone **1** (460 mg, 1 mmol, 1 equiv), aniline (931 mg, 10 mmol, 10 equiv), DABCO (673 mg, 6 mmol, 6 equiv) was added PhCl (20 mL) under nitrogen atmosphere. The two-neck bottle was heated to 60 °C with an oil bath, followed by slow addition of $TiCl_4$ (0.67ml, 6 mmol, 6 equiv) at 60 °C. The reaction mixture was stirred under 140 °C for 2 days (or more time, see Table 2 for details). After the reaction mixture was cooled to rt, the reaction mixture was poured into 100 mL of DCM, quenched by $NaHCO_3$(aq), and extracted with DCM (3 × 50 mL). In cases there exist undissolved solid residues, the solid residue was filtered and washed with DCM/MeOH cosolvent (DCM/MeOH = 10/1) or THF, and the organic solution was combined with the DCM layer. The combined organic solution was dried over $MgSO_4$ and then concentrated. The resulting crude product was purified by recrystallization or column chromatography (solvent system for each case is shown independently in section 2.2) using silica gel to afford the isolated yield as the yield of one-pot synthesis.

For two-step synthesis (steps 1 and 2): The step 1 procedure is the same as the case of one-pot synthesis described above, and the resulting crude product was directly used for the step 2, as described in the following. To a bottle equipped with a stirring bar containing the crude product of step 1 was added 30 mL THF/MeOH cosolvent (THF/MeOH = 3/1) under air atmosphere. The mixture was cooled to 0 °C, followed by the addition of excess $NaBH_4$ (189 mg, 5 mmol, 5 equiv). The mixture was stirred under rt for 30 min, and then quenched by ice-water. The THF/MeOH cosolvent was removed by a rotavapor. The residue was then extracted with DCM or THF (3 × 50 mL). The combined organic layer was washed with brine and dried over by $MgSO_4$ and concentrated. The crude product was purified by the same method for the one-pot synthesis (solvent system for each case is shown independently in section 2.2).

2.2 Purification method and characterization data of 2a-2t

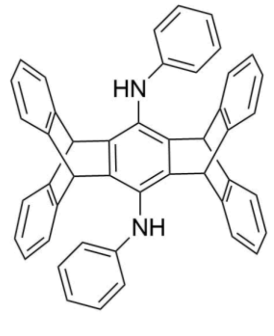

2a, R_f = 0.46 (DCM/Hexane = 1/2), The reaction mixture was purified by purified by column chromatography (DCM/Hexane = 1/2 to 1/1) to afford **2a** as yellow solid. 88% yield (538 mg) for one-pot, 91% yield (559 mg) for 2-step procedure. Mp: >300 °C. ^{1}H NMR (400 MHz, $CDCl_3$) ^{1}H NMR (400 MHz, $CDCl_3$) δ 7.25 – 7.17 (m, 4H), 7.17 – 7.06 (m, 8H), 6.92 – 6.83 (m, 10H), 6.59 – 6.52 (m, 4H), 5.54 (s, 4H). **^{13}C NMR** (126 MHz, $CDCl_3$) δ 147.4, 145.1, 141.9, 129.5 (2C), 125.0, 123.7, 118.6, 114.2, 77.2, 49.6. **HRMS** (ESI) m/z : $[M+H]^+$ Calcd for $C_{46}H_{33}N_2$ 613.2638; found 613.2611

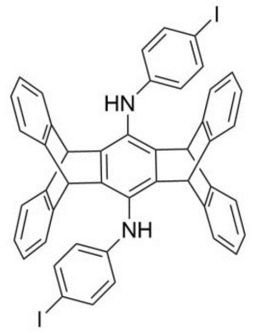

2b, R_f = 0.54 (DCM/Hexane = 1/1). In this case, the starting material 4-Iodoaniline was recrystallized from hexane to afford white crystal before use. The crude product was purified by column chromatography (DCM/Hexane = 1/4 to 1/2) to afford **2b** as beige solid. 77% (662 mg) for one-pot; 80% (691 mg) yield for 2-step procedure; Mp: >300 °C. **^{1}H NMR** (400 MHz, CD_2Cl_2) δ 7.49 – 7.43 (m, 4H), 7.17 – 7.09 (m, 8H), 6.96 – 6.85 (m, 8H), 6.35 – 6.29 (m, 4H), 5.71 (s, 2H), 5.52 (s, 4H). **^{13}C NMR** (126 MHz, CD_2Cl_2) δ 147.7, 145.5, 142.7, 138.6, 129.6, 125.7, 124.3, 116.8, 79.9, 50.1. **HRMS** (ESI) m/z: $[M+H]^+$ Calcd for $C_{46}H_{31}I_2N_2$ 865.0571; found 865.0597.

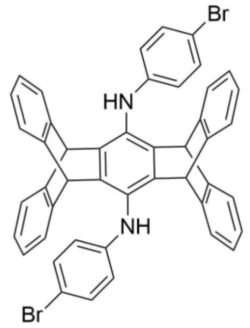

2c, R_f = 0.4 (DCM/Hexane = 1/2). The crude product was purified by column chromatography (DCM/Hexane = 1/1) to afford **2c** as off-white solid. 89% yield (683 mg) for one-pot and 91% yield (694 mg) for 2-step procedure; Mp: >300 °C. **^{1}H NMR** (400 MHz, CD_2Cl_2) δ 7.33 – 7.25 (m, 4H), 7.17 – 7.09 (m, 8H), 6.94 – 6.86 (m, 8H), 6.46 – 6.37 (m, 4H), 5.70 (s, 2H), 5.53 (s, 4H). **^{13}C NMR**

(126 MHz, CD_2Cl_2) δ 147.1, 145.5, 142.7, 132.7, 129.7, 125.7, 124.3, 116.2, 110.7, 50.1. **HRMS** (ESI) m/z: $[M+H]^+$ Calcd for $C_{46}H_{31}Br_2N_2$ 769.0848; found 769.0866.

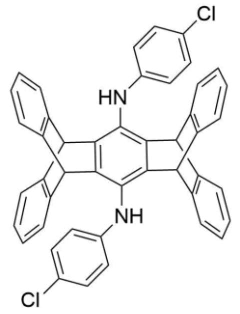

2d, R_f = 0.41 (DCM/Hexane = 1/1.5). The crude product was purified by column chromatography (DCM/Hexane = 1/2) to afford **2d** as beige solid. 88% yield (598 mg) for one-pot procedure and 90% yield (609 mg) for 2-step procedure; Mp: >300 °C. **^{1}H NMR** (500 MHz, CD_2Cl_2) δ 7.19 – 7.10 (m, 12H), 6.93 – 6.86 (m, 8H), 6.49 – 6.42 (m, 4H), 5.70 (s, 2H), 5.53 (s, 4H), 5.40 – 5.28 (m, 2H), 5.33 (s, 1H). **^{13}C NMR** (126 MHz, CD_2Cl_2) δ 146.7, 145.6, 142.7, 129.8, 125.7, 124.6, 123.6, 115.7, 50.1. **HRMS** (ESI) m/z: $[M+H]^+$ Calcd for $C_{46}H_{31}Cl_2N_2$ 681.1859; found 681.1882.

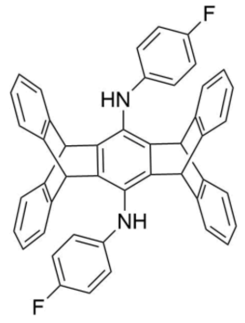

2e, R_f = 0.36 (DCM/Hexane = 1/2). The crude product was purified by recrystallization from DCM and hexane after column chromatography (DCM/Hexane = 1/3 to 1/1) to afford **2e** as beige solid. 67% yield (431 mg) for one-pot and 69% yield (444 mg) for 2-step procedure (Note: because of the additional recrystallization step, the yield is somewhat reduced as compared with **2c** and **2d**); Mp: >300 °C. **^{1}H NMR** (500 MHz, CD_2Cl_2) δ 7.16 – 7.09 (m, 8H), 6.96 – 6.85 (m, 12H), 6.50 – 6.42 (m, 4H), 5.60 (s, 2H), 5.54 (s, 4H). **^{13}C NMR** (126 MHz, CD_2Cl_2) δ 158.0, 156.1, 145.7, 144.5 (2C), 142.5, 130.4, 125.6, 124.2, 116.4, 116.3, 115.7, 115.6, 50.1. **^{19}F NMR** (471 MHz, CD_2Cl_2) δ 128.35**.** **HRMS** (ESI) m/z: $[M+H]^+$ Calcd for $C_{46}H_{31}F_2N_2$ 649.2450; found 649.2417.

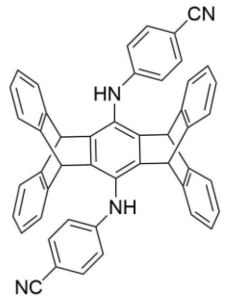

2f, R_f = 0.35 (DCM/Hexane = 2/1); This product shows poor solubility. The crude product was poured into DCM, and the undissolved solid residue was filtered and washed by THF (2×50 mL). The combined organic layer was dried over $MgSO_4$, then concentrated, and purified by column chromatography (THF/Hexane = 1/3 to pure THF) to afford **2f** as brown solid. 50% yield (307 mg) for one pot at 2 d, 60% yield (396 mg) for one-pot at 4d, and 61% yield (404 mg) for 2-step procedure; Mp: >300 °C. **^{1}H NMR** (500 MHz, Acetone-d_6) δ 8.27 (s, 2H), 7.54 – 7.49 (m, 4H), 7.21 – 7.14 (m, 8H), 6.92 – 6.84 (m, 8H), 6.57 (d, J = 6.8 Hz, 4H), 5.66 (s, 4H). **^{13}C NMR** (126 MHz, Acetone-d_6) δ 153.1, 146.6, 143.6, 135.0, 130.0, 126.3, 125.1, 121.1, 115.0, 101.0, 50.9. **HRMS** (ESI) m/z: $[M+H]^+$ Calcd for $C_{48}H_{31}N_4$ 663.2543; found 663.2514.

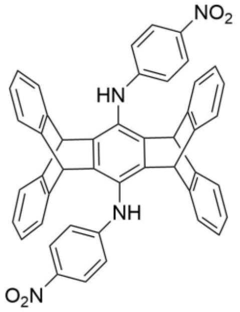

2g, R_f = 0.16 (DCM/Hexane = 2/1), The reaction mixture was purified by column chromatography (DCM/Hexane = 1/1 to pure DCM) to afford 627 mg of **2g** as a yellow solid. 72% (504 mg) for one-pot at 2 d, 84% (587 mg) for one-pot at 4 d, and 87% (627 mg) yield for 2-step procedure; Mp: >300 °C. **^{1}H NMR** (500 MHz, CD_2Cl_2) δ 8.12 (d, J = 8.8 Hz, 4H), 7.17 – 7.13 (m, 8H), 6.94 – 6.90 (m, 8H), 6.53 (d, J = 8.6 Hz, 4H), 6.35 (s, 2H), 5.51 (s, 4H). **^{13}C NMR** (126 MHz, CD_2Cl_2) δ 153.2, 145.1, 143.4, 140.2, 128.7, 127.0, 126.0, 124.4, 113.3, 50.1. **HRMS** (ESI) m/z: $[M+H]^+$ Calcd for $C_{46}H_{31}N_4O_4$ 703.2340; found 703.2328

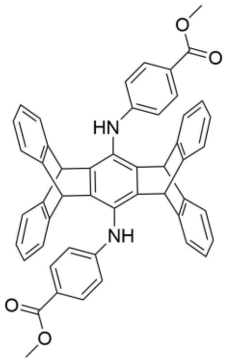

2h, R_f = 0.55 (DCM/Hexane = 3/1). The crude product was purified by column chromatography (DCM/Hexane = 1/1 to pure DCM) to afford **2h** as white solid. 80% yield (585 mg) for one-pot and 84% yield (609 mg) for 2-step procedure; Mp: >300 °C. **^{1}H NMR** (500 MHz, CD_2Cl_2) δ 7.92 (d, J = 8.4 Hz, 4H), 7.20 – 7.14 (m, 8H), 6.97 – 6.90 (m, 8H), 6.55 (d, J = 8.4 Hz, 4H), 6.09 (s, 2H), 5.57 (s, 4H), 5.36 (s, 4H), 3.92 (s, 6H). **^{13}C NMR** (126 MHz, CD_2Cl_2) δ 167.5, 151.8, 145.4, 143.0,

132.1, 129.2, 125.7, 124.3, 120.8, 113.5, 52.1, 50.1. **HRMS** (ESI) m/z: $[M+H]^+$ Calcd for $C_{50}H_{37}N_2O_4$ 729.2748; found 729.2712.

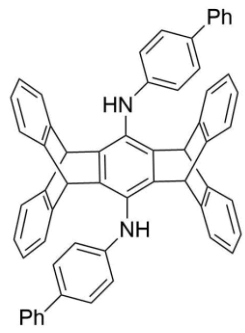

2i, R_f = 0.26 (DCM/Hexane = 1/2), The crude product was purified by column chromatography (DCM/Hexane = 1/2) to afford **2i** as a reddish solid. 62% yield (472 mg) for one-pot and 65% yield (493 mg) for 2-step procedure; Mp: >300 °C. ^{1}H NMR (400 MHz, CD_2Cl_2) δ 7.67 – 7.60 (m, 4H), 7.54 – 7.40 (m, 8H), 7.35 – 7.26 (m, 2H), 7.17 (m, 8H), 6.90 (m, 8H), 6.68 – 6.59 (m, 4H), 5.62 (s, 4H). **^{13}C NMR** (126 MHz, $CDCl_3$) δ 146.9, 145.1, 141.9, 141.2, 131.5, 129.4, 128.8, 128.2, 126.4, 126.3, 125.1, 123.8, 114.5, 49.6. **HRMS** (ESI) m/z: $[M+H]^+$ Calcd for $C_{58}H_{41}N_2$ 765.3264; found 765.3229.

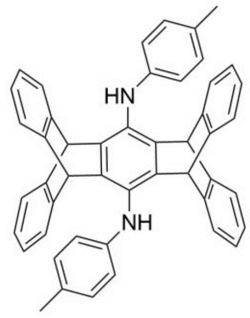

2j, R_f = 0.4 (DCM/Hexane = 1/2). The crude product was purified by column chromatography (DCM/Hexane = 1/3 to 1/2) to afford **2j** as beige solid. 78% yield (500 mg) for one-pot and 83% yield (527 mg) for 2-step procedure; Mp: >300 °C. **^{1}H NMR** (400 MHz, CD_2Cl_2) δ 7.17 – 7.08 (m, 8H), 7.06 – 6.96 (m, 4H), 6.92 – 6.83 (m, 8H), 6.49 – 6.41 (m, 4H), 5.57 – 5.52 (m, 6H), 5.35 – 5.27 (m, 1H), 2.33 (s, 6H). **^{13}C NMR** (126 MHz, CD_2Cl_2) δ 145.9, 145.8, 142.1, 130.4 (2C), 128.4, 125.5, 124.2, 114.8, 50.1, 20.8. **HRMS** (ESI) m/z: $[M+H]^+$ Calcd for $C_{48}H_{37}N_2$ 641.2951; found 641.2930.

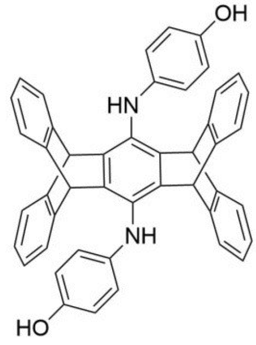

2k, R_f = 0.3 (DCM/Hexane = 2/1). This product shows poor solubility in DCM. The crude product was poured into DCM, and the undissolved solid residue was filtered and washed by THF (3×50 mL). The combined organic layer was dried over $MgSO_4$, then concentrated, and purified by column chromatography (pure DCM to MeOH/DCM = 1/9) to afford **2k** as dark-blue solid. 38% (245 mg) for one-pot and 42% yield (271 mg) for 2-step procedure; Mp: >300 °C. **^{1}H**

NMR (400 MHz, Acetone-d_6) δ 7.66 (s, 2H), 7.16 – 7.08 (m, 8H), 6.88 – 6.79 (m, 10H), 6.69 – 6.65 (m, 4H), 6.46 – 6.42 (m, 4H), 5.70 (d, J = 0.7 Hz, 4H). **^{13}C NMR** (126 MHz, Acetone-d_6) δ 151.4 (2C), 151.3, 147.3, 142.8 (2C), 142.1 (2C), 138.2, 138.1, 126.0, 125.0, 117.2, 117.1, 50.9. **HRMS** (ESI) m/z: $[M+H]^+$ Calcd for $C_{46}H_{33}N_2O_2$ 645.2537; found 645.2524

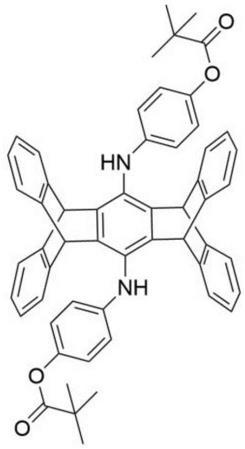

2l, R_f = 0.6 (DCM/Hexane = 1/1). The reaction mixture was purified column chromatography (DCM/Hexane = 1/3) to afford **2l** as blown solid. 53% yield (404 mg) for one-pot and 56% yield (457 mg) for 2-step procedure; Mp: >300 °C. **^{1}H NMR** (400 MHz, CD_2Cl_2) δ 7.23 – 7.14 (m, 8H), 6.97 – 6.89 (m, 12H), 6.60 – 6.52 (m, 4H), 5.70 (s, 2H), 5.61 (s, 4H), 1.41 (s, 18H) . **^{13}C NMR** (126 MHz, CD_2Cl_2) δ 178.2, 145.8, 145.7, 144.1, 142.5, 130.3 125.6, 124.3, 122.9, 115.1, 50.1, 39.5, 27.4. **HRMS** (ESI) m/z: $[M+H]^+$ Calcd for $C_{56}H_{49}N_2O_4$ 813.3687; found 813.3672.

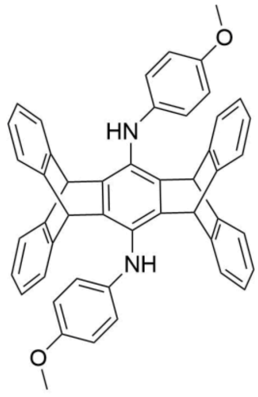

2m, R_f = 0.6 (DCM/Hexane = 1/1). The starting material *p*-anisidine is required to be recrystallized from hot EtOH to afford reddish crystal. The reaction mixture was purified by column chromatography (DCM/Hexane = 1/3 to 1/1) to afford of **2m** as pink solid, 19% yield (126 mg) for one-pot. 23% yield (156 mg) for 2-step procedure; Mp: >300 °C. **^{1}H NMR** (400 MHz, CD_2Cl_2) δ 7.16 – 7.08 (m, 8H), 6.92 – 6.85 (m, 8H), 6.82 – 6.75 (m, 4H), 6.53 – 6.46 (m, 4H), 5.56 (s, 4H), 5.48 (s, 2H), 3.80 (s, 6H). **^{13}C NMR** (126 MHz, CD_2Cl_2) δ 153.6, 145.9, 142.2, 141.9, 130.8, 125.5, 124.2, 116.2, 115.4, 56.3, 50.0. **HRMS** (ESI): m/z: $[M+H]^+$ Calcd for $C_{48}H_{37}N_2O_2$ 673.2850; found 673.2837

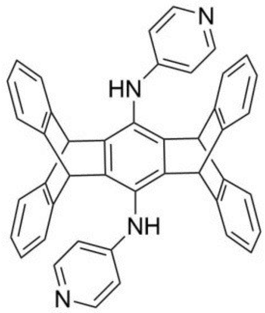

2n, R_f = 0.34 (MeOH/DCM = 1/20). This product shows poor solubility. The crude product was poured into THF, and the undissolved solid residue was filtered and washed by MeOH/DCM cosolvent (3×50 ml, MeOH/DCM = 1/10). The combined organic layer was dried over $MgSO_4$, then concentrated. The residue was washed by MeOH to afford **2n** as beige solid. 75% yield (463 mg) for one-pot and 76% yield (466 mg) for 2-step procedure; Mp: >300 °C. The ^{13}C NMR spectrum was collected by CD_2Cl_2/CD_3OD = 5/1 due to the solubility issue, The solvent peak of CD_2Cl_2 located in 54.00 ppm. **^{1}H NMR** (400 MHz, Acetone-*d*$_6$) δ 8.18 (d, *J* = 5.6 Hz, 4H), 7.22 – 7.14 (m, 8H), 6.93 – 6.84 (m, 8H), 6.44 – 6.35 (m, 4H), 5.67 (s, 2H). **^{13}C NMR** (126 MHz, CD_2Cl_2/CD_3OD = 5/1) δ 154.7, 149.9, 145.5, 142.9, 128.5, 125.8, 124.3, 109.4, 50.2. **HRMS** (ESI) m/z: $[M+H]^+$ Calcd for $C_{44}H_{31}N_4$ 615.2543; found 615.2513

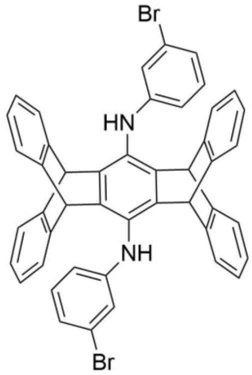

2o, R_f = 0.43 (DCM/Hexane = 1/1). The crude product was purified by column chromatography (DCM/Hexane = 1/1) to afford **2o** as beige solid. 90% yield (689 mg) for one-pot and 93% yield (714 mg) for 2-step procedure; Mp: >300 °C. **^{1}H NMR** (400 MHz, CD_2Cl_2) δ 7.20 – 7.12 (m, 8H), 7.09 (t, *J* = 7.9 Hz, 2H), 7.00 (dd, *J* = 7.9, 1.6 Hz, 2H), 6.95 – 6.87 (m, 8H), 6.65 (m, 2H), 6.49 (dd, *J* = 7.9, 2.2 Hz, 2H), 5.74 (s, 2H), 5.53 (s, 4H). **^{13}C NMR** (126 MHz, CD_2Cl_2) δ 149.3, 145.5, 142.6, 131.5, 129.4, 125.7, 124.3, 124.0, 122.0, 116.9, 113.4, 50.1. **HRMS** (ESI) m/z: $[M+H]^+$ Calcd for $C_{46}H_{31}Br_2N_2$ 769.0848; found 769.0817.

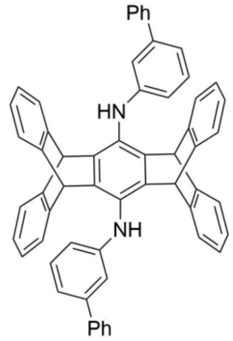

2p, R_f = 0.53 (DCM/Hexane = 1/1). The crude product was purified by column chromatography (DCM/Hexane = 1/3 to 1/2) to afford **2p** as a yellow-green solid. 71% yield (541 mg) for one-pot and 74% yield (556 mg) for 2-step procedure; Mp: >300 °C. **^{1}H NMR** (400 MHz, CD_2Cl_2) δ 7.53 – 7.45 (m, 4H), 7.41 – 7.22 (m, 7H), 7.28 – 7.24 (m, 2H), 7.17 – 7.08 (m, 10H), 6.88 – 6.77 (m, 10H), 6.51 – 6.42 (m, 2H), 5.76 (s, 2H), 5.62 (s, 4H). **^{13}C NMR** (126 MHz, CD_2Cl_2) δ 148.6, 145.8, 143.1, 142.6, 141.9, 130.5, 130.2, 129.1, 127.8, 127.6, 125.5, 124.3, 118.1, 113.8, 113.4, 50.1. **HRMS** (ESI) m/z: $[M+H]^+$ Calcd for $C_{58}H_{41}N_2$ 765.3264; found 765.3277.

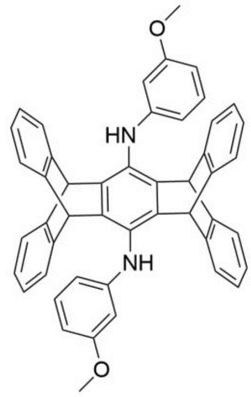

2q, R_f = 0.55 (DCM/Hexane = 1/1). The crude product was purified by column chromatography (DCM/Hexane = 1/2 to 1/1) to afford **2q** as off-white solid. 64 % yield (430 mg) for one-pot procedure and 66% yield (446 mg) for 2-step procedure; Mp: >300 °C. **^{1}H NMR** (500 MHz, CD_2Cl_2) δ 7.17 – 7.06 (m, 10H), 6.92 – 6.84 (m, 8H), 6.43 (dd, *J* = 8.1, 2.3, 0.8 Hz, 2H), 6.12 (ddd, *J* = 8.1, 2.3, 0.8 Hz, 2H), 6.01 (t, *J* = 2.3 Hz, 2H), 5.67 (s, 2H), 5.56 (s, 4H), 3.61 (s, 6H). **^{13}C NMR** (126 MHz, CD_2Cl_2) δ 161.6, 149.5, 145.8, 142.5, 130.8, 130.1, 125.5, 124.3, 107.6, 104.6, 100.7, 55.5, 50.1. **HRMS** (ESI) m/z: $[M+H]^+$ Calcd for $C_{48}H_{37}N_2O_2$ 673.2850; found 673.2843.

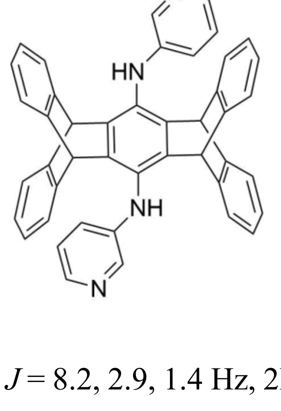

2r, R_f = 0.2 (MeOH/DCM = 1/20). The same purification method as the case of **2n** was adopted to afford **2r** as beige solid. 67% yield (411 mg) for one-pot and 71% yield (434 mg) for 2-step procedure; Mp: >300 °C. **^{1}H NMR** (500 MHz, DMSO-d_6) δ 8.21 (s, 2H), 7.97 (dd, *J* = 4.5, 1.4 Hz, 2H), 7.91 (m, 2H), 7.16 – 7.11 (m, 8H), 7.09 (dd, *J* = 8.2, 4.5 Hz, 2H), 6.87 (m, 8H), 6.55 (ddd, *J* = 8.2, 2.9, 1.4 Hz, 2H), 5.54 (s, 4H). **^{13}C NMR** (126 MHz, DMSO-d_6) δ 145.0, 144.2, 141.0, 138.7, 136.3, 128.8 (2C), 123.7 (2C), 119.2, 48.8. **HRMS** (ESI) m/z: $[M+H]^+$ Calcd for $C_{44}H_{31}N_4$ 615.2543; found 615.2531.

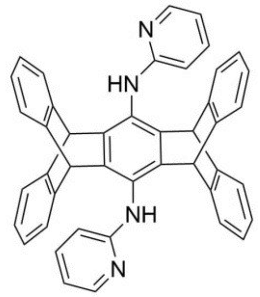

2s, R_f = 0.36 (MeOH/DCM = 1/20). The same purification method as the case of **2n** was adopted to afford **2p** as off-white solid. However, an additional step was performed: before the MeOH washing, column chromatography (short column with silica gel, MeOH/DCM = 1/10) to remove extra 2-aminopyridine is required. 71% yield (439 mg) for one-pot and 73% yield (446 mg) for 2-step procedure; Mp: >300 °C. The ^{13}C NMR spectrum was collected by CD_2Cl_2/CD_3OD = 5/1 due to the solubility issue, The solvent peak of CD_2Cl_2 located in 54.00 ppm. **^{1}H NMR** (400 MHz, DMSO-d_6) δ 8.72 (s, 2H), 7.97 – 7.91 (m, 2H), 7.54 – 7.46 (m, 2H), 7.14 (m, 3.2 Hz, 8H), 6.93 – 6.82 (m, 8H), 6.76 – 6.69 (m, 2H), 6.42 (d, *J* = 8.4 Hz, 2H), 5.48 (s, 4H). **^{13}C NMR** (126 MHz, CD_2Cl_2/CD_3OD = 5/1) δ 159.4, 148.6, 145.6, 142.9, 139.2, 128.8, 125.7, 124.3, 115.0, 108.1, 50.2. **HRMS** (ESI) m/z: $[M+H]^+$ Calcd for $C_{44}H_{31}N_4$ 615.2543; found 615.2520.

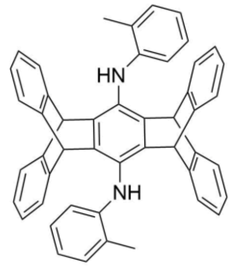

2t, R_f = 0.54 (DCM/Hexane = 1/3). The reaction time for step 1 is 5 days. The crude product was purified by column chromatography (DCM/Hexane = 1/4 to 1/3) to afford **2t** as a brown solid. 18% yield (116 mg) for one-pot and 20% yield (127 mg) for 2-step procedure; Mp: >300 °C. **^{1}H NMR** (400 MHz, CD_2Cl_2) δ 7.38 – 6.77 (m, 22H) , 5.91 – 5.78 (m, 2H), 5.46 (s, 4H), 2.61 (s, 6H). **^{13}C NMR** (126 MHz, CD_2Cl_2) δ 146.27, 145.8, 142.4, 131.0, 130.7, 127.8, 125.5, 124.2, 122.9, 119.1, 114.0, 50.1, 18.4. **HRMS** (ESI) m/z: $[M+H]^+$ Calcd for $C_{48}H_{37}N_2$ 641.2951; found 641.2946

2.3 Procedures for the derivatization of 2c (synthesis of 2v, 2w, 2i and 4)

Heck reaction:

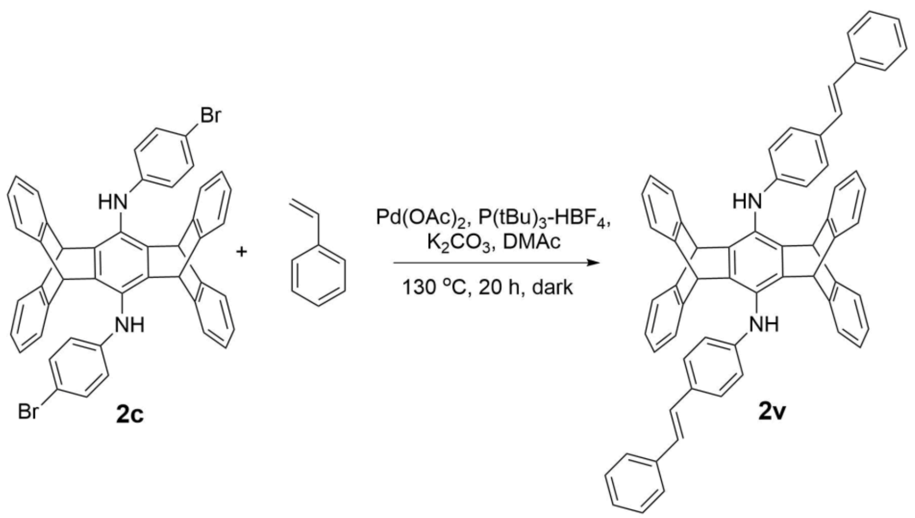

Synthesis of 2v. To an oven-dried Schlenk tube were added with **2c** (154 mg, 0.2 mmol, 1.0 equiv), K_2CO_3 (221 mg, 1.6 mmol, 4.0 equiv), and $Pd(OAc)_2$ (9 mg, 0.04 mmol, 0.2 equiv). 1.2 mL degassed *N,N*-dimethylacetamide (DMAc) was then added, followed by the addition of a solution of 0.1 mM HP-(*t*-Bu)$_3$BF$_4$ solution in DMAc (0.8 ml, 0.08 mmol, 0.4 equiv) in DMAc bringing the reaction concentration to 0.1 M. The mixture was then purged with nitrogen for 10 min. The reaction mixture was allowed to stir at room temperature for 5 min. Styrene (42 mg, 0.4 mmol, 2.0 equiv) was then added, and the reaction temperature was raised to 130 °C with an oil bath. After 20 h, the reaction mixture was filtered through a short pad of Celite using DCM as eluent. The filtrate was concentrated under reduced pressure, and 20 mL water be added to cause precipitation. The precipitate was washed with brine and cold methanol. The residue was then purified via column chromatography (DCM/Hexane = 1:3 to 1/1) to afford **2v** as a yellow solid. 76% yield (125 mg); R_f = 0.36 (DCM/Hexane = 1/1.5) Mp: 298.1-299.3 ºC (decomp.). **^{1}H NMR** (500 MHz, CD_2Cl_2) δ 7.56 – 7.51 (m, 4H), 7.41 (d, *J* = 8.5 Hz, 4H), 7.36 (t, *J* = 7.7 Hz, 4H), 7.27 – 7.19 (m, 2H), 7.19 – 7.11 (m, 10H), 7.01 (d, *J* = 16.2 Hz, 2H), 6.90 (dd, *J* = 5.5, 3.1 Hz, 8H), 6.57 – 6.51 (m, 4H), 5.80 (s, 2H), 5.58 (s, 4H). **^{13}C NMR** (126 MHz, CD_2Cl_2) δ 147.8, 145.7, 142.6, 138.6, 129.9, 129.2 (2C), 128.6, 128.5, 127.5, 126.6, 125.6, 125.5, 124.3, 114.8, 50.1. **HRMS** (ESI) m/z: $[M+H]^+$ Calcd for $C_{62}H_{45}N_2$ 817.3577; found 817.3557

Sonogashira reaction:

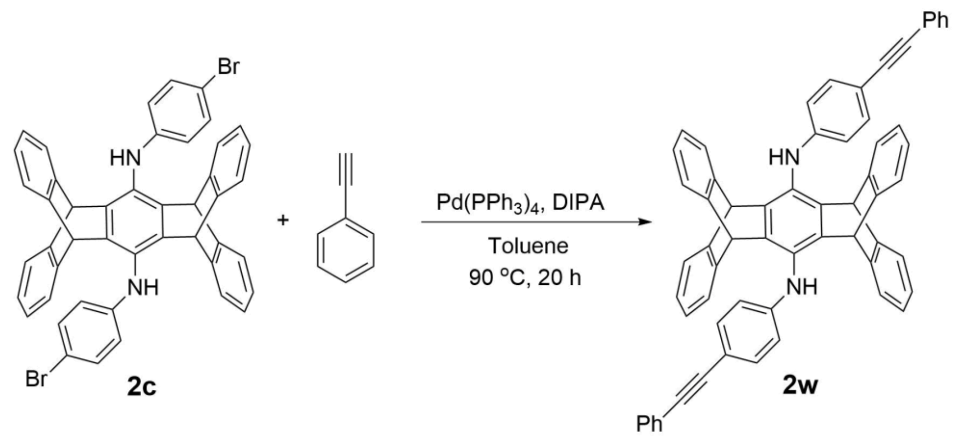

Synthesis of 2w. A mixture of **2c** (77 mg, 0.1 mmol, 1 equiv) and $Pd(PPh_3)_4$ (35 mg, 0.03 mmol, 0.3 equiv) in a Schlenk flask under nitrogen were added with phenylacetylene (49 mg, 0.25 mmol, 2.5 equiv) and 2.1 mL diisopropylamine and 2.1 mL toluene and then heated to 90 ºC with an oil bath for 20 h. The mixture was passed through celite and concentrated under reduced pressure. The residue was extracted in DCM and water, then concentrated under reduced pressure. Column chromatography (DCM/Hexane from 1/5 to 1/1) afforded **2x** as a brown-yellow solid. 80% yield (52 mg); Rf = 0.26 (DCM/Hexane = 1/1.2), Mp = 232.9-234.7 ºC (decomp.). **^{1}H NMR** (400 MHz, DMSO-d_6) δ 8.13 (s, 2H), 7.65 (d, J = 7.6 Hz, 4H), 7.51 – 7.40 (m, 8H), 7.29 (t, J = 7.4 Hz, 2H), 7.22 – 7.15 (m, 8H), 6.95 – 6.86 (m, 8H), 6.57 (d, J = 8.2 Hz, 4H), 5.60 (s, 4H). **^{13}C NMR** (126 MHz, $CDCl_3$) δ 147.3, 144.8, 142.2, 133.3, 131.4, 128.8, 128.3, 127.8, 125.2, 123.9, 123.8, 113.8, 112.8, 90.2, 87.7, 49.6. **HRMS** (ESI) m/z: $[M+H]^+$ Calcd for $C_{62}H_{41}N_2$ 813.3264; found 813.3276.

Suzuki reaction:

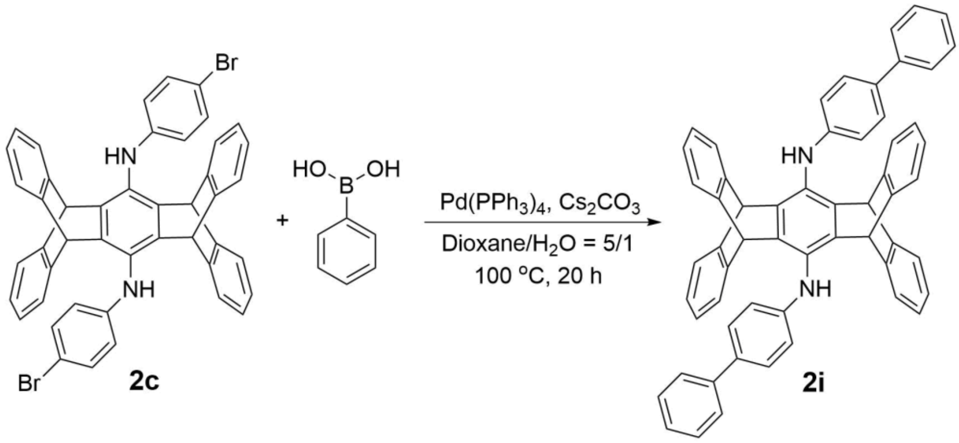

Synthesis of 2i, A mixture of **2c** (77 mg, 0.1 mmol, 1 equiv), $Pd(PPh_3)_4$ (35 mg, 0.03 mmol, 0.3 equiv), phenylboronic acid (30 mg, 0.25 mmol, 2.5 equiv), cesium carbonate (114 mg, 0.35 mmol, 3.5 equiv), 1 mL H_2O, and 5 mL dioxane in Schlenk flask under nitrogen was heated to 100 ºC with an oil bath for 20 h. The mixture was passed through celite and concentrated under reduced pressure. The residue was extracted in DCM and water, then the organic layer was concentrated under reduced pressure. Column chromatography (DCM/Hexane from 1/10 to 1/1) afforded **2i** as a reddish solid. 95% yield (72 mg); Mp > 300 ºC. See section 2.2 for characterization data.

S_NAr reaction

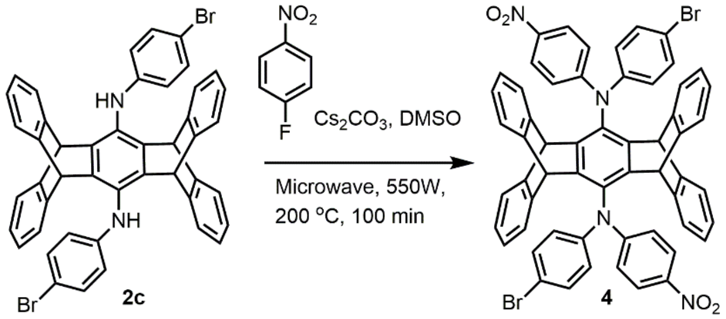

Synthesis of 4. A mixture of **2c** (154 mg, 0.2 mmol, 1 equiv), 1-fluoro-4-nitrobenzene (71 mg, 0.5 mmol, 5 equiv), cesium carbonate (163 mg, 0.5 mmol, 5 equiv), and 5 mL of DMSO were placed in a flask. The open flask was then radiated in a microwave oven with the flask open a power output of 550 W (temperature set to 200°C,) for 50 min. To avoid splashing during the reaction, we ran this reaction on a smaller scale (i.e., 0.2 mmol instead of 1.0 mmol). Then, a second batch of 1-fluoro-4-nitrobenzene (71 mg, 0.5 mmol, 5 equiv) was added to the mixture. A second round of microwave irradiation under the same conditions mentioned above was carried out. After cooling, the reaction mixture was extracted with DCM and water, and the organic layer was concentrated under reduced pressure. The residue was purified by column chromatography (DCM/Hexane = 3/1) to afford **4** as a yellow solid. 90% yield (182 mg); Rf = 0.2 (DCM/Hexane = 3:1). Mp > 300 °C. ^{1}H NMR (500 MHz, DMSO-d_6) δ 8.18 – 8.08 (m, 4H), 7.67 – 7.57 (m, 4H), 7.19 – 7.09 (m, 4H), 6.88 – 6.77 (m, 16H), 6.76 – 6.65 (m, 4H), 5.44 – 5.40 (m, J = 1.9 Hz, 4H). ^{13}C NMR (126 MHz, DMSO-d_6) δ 152.5, 143.9, 143.8 (2C), 143.8, 143.7, 143.6, 140.1, 133.4, 133.0 (2C), 126.3, 126.2, 126.1 (2C), 125.2 (2C), 123.7, 123.6, 123.2, 123.1, 117.2, 117.2, 117.0 (2C), 48.8. **HRMS** (ESI) m/z: $[M+H]^+$ Calcd for $C_{58}H_{37}Br_2N_4O_4$ 1011.1176; found 1011.1166

3. Spectra

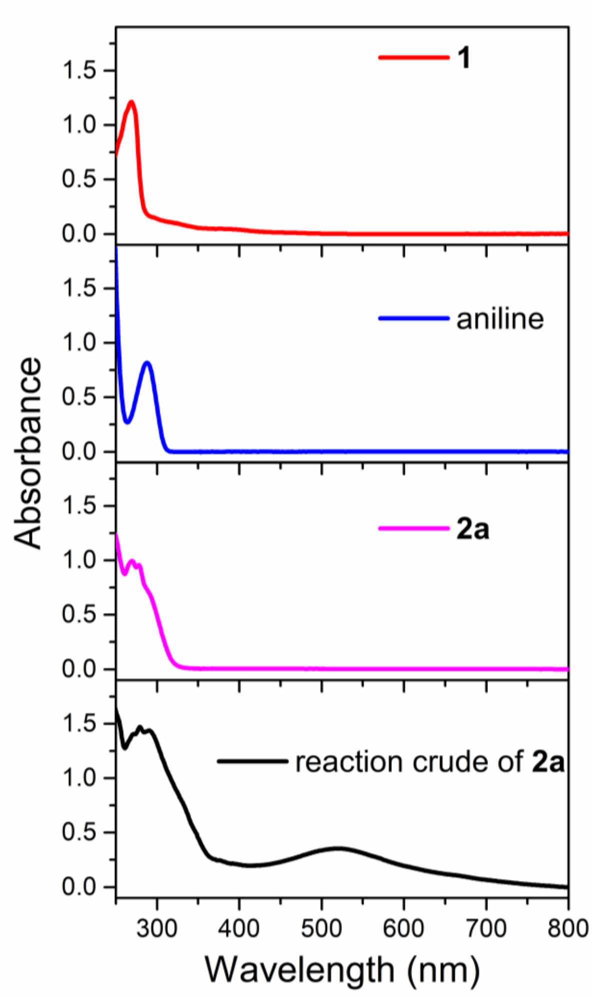

Figure S1. UV-vis absorption spectra of **1**, aniline, **2a**, and crude product of **2a**. The spectrum of the reaction crude resembles that of pernigraniline base (PB), an oxidized form of polyaniline.[S5]

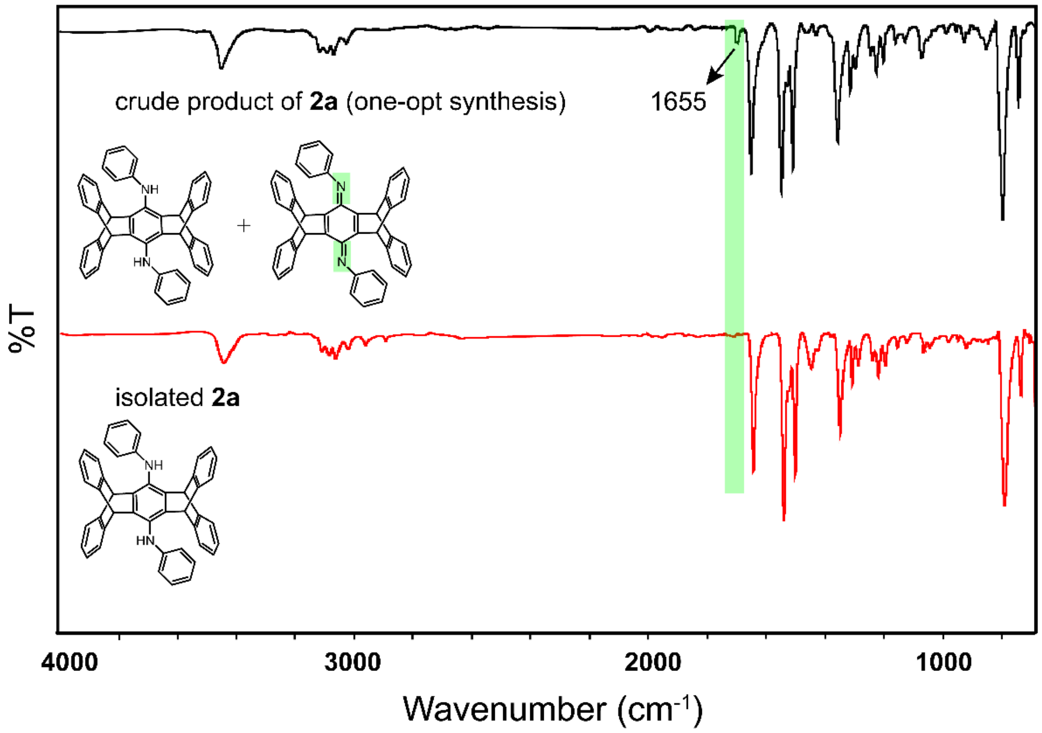

Figure S2. FT-IR spectra of crude product of **2a** from one-pot synthesis (upper spectrum) and the isolated **2a** (bottom spectrum).

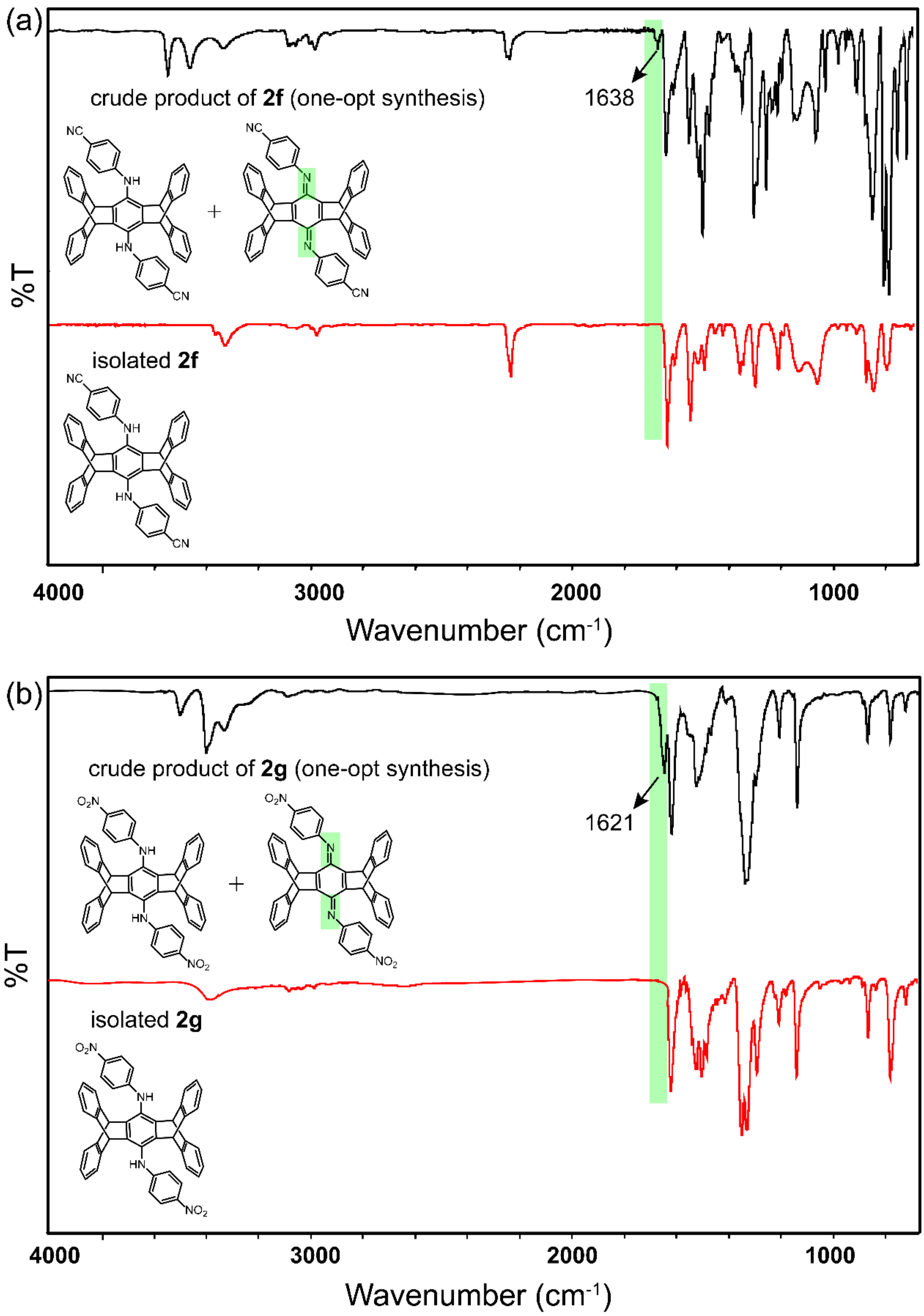

Figure S3. FT-IR spectra of (a) crude product of **2f** from one-pot synthesis (upper spectrum) and the isolated **2f** (bottom spectrum) and (b) crude product of **2g** from one-pot synthesis (upper spectrum) and the isolated **2g** (bottom spectrum).

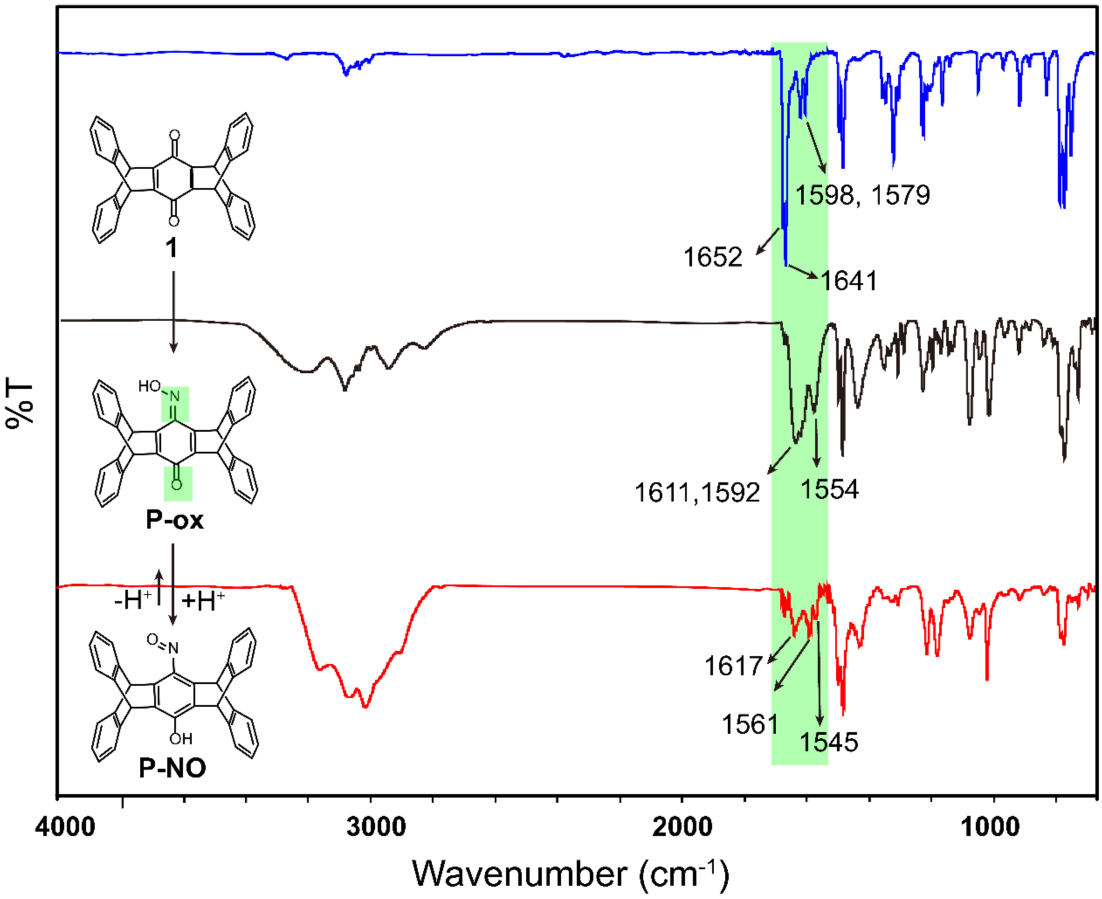

Figure S4. FT-IR spectra of **1** (upper), **P-ox** (middle) and **P-NO** (bottom).

Note: **P-NO** was prepared by dissolving **P-ox** (90 mg) in 20 mL of THF (HPLC grade) in the presence of 2 mL HCl aqueous solution (37%).

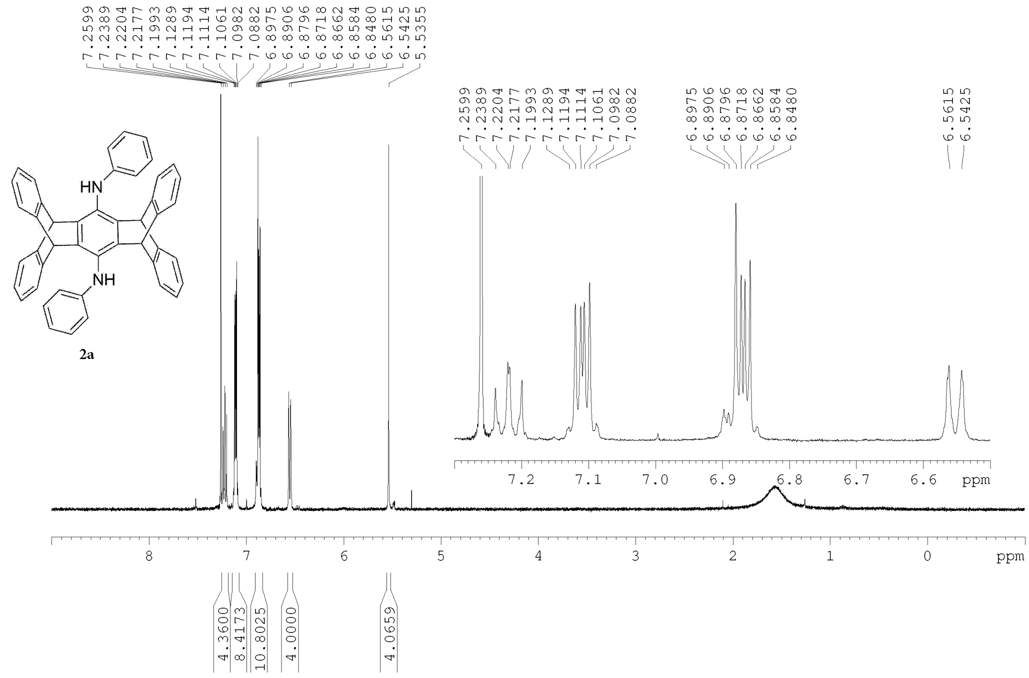

Figure S5. ^{1}H spectrum of compound **2a** ($CDCl_3$, 400 MHz)

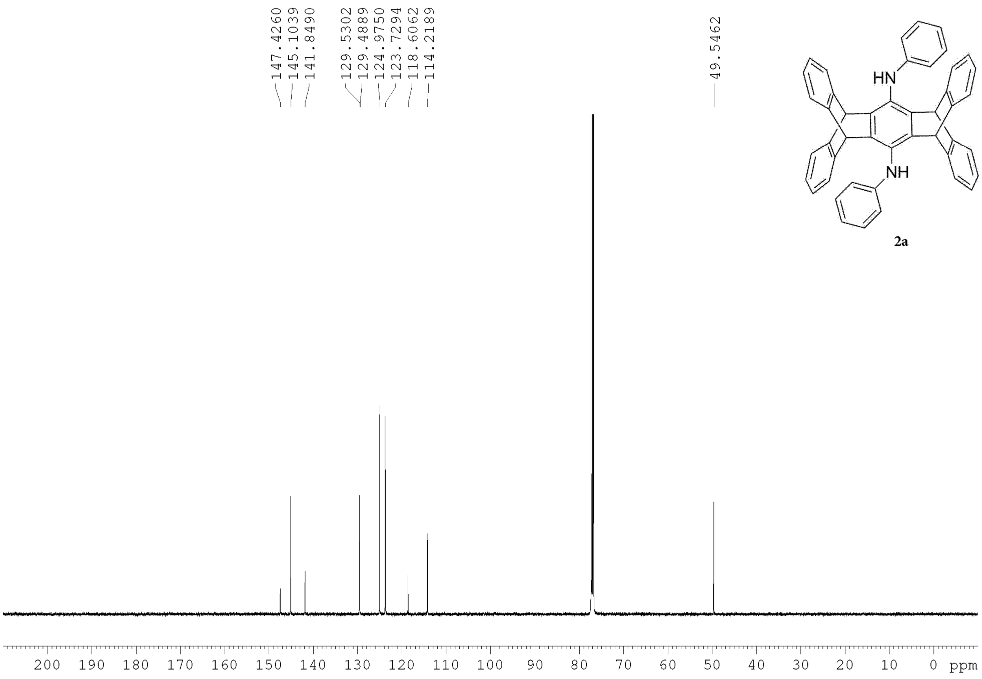

Figure S6. ^{13}C spectrum of compound **2a** ($CDCl_3$, 126 MHz)

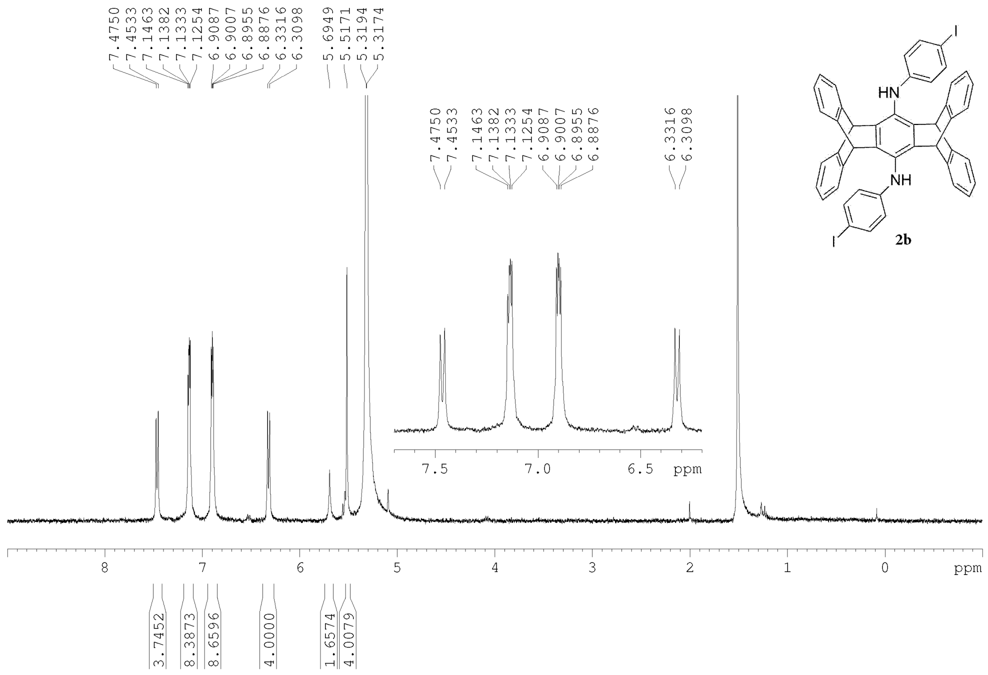

Figure S7. ^{1}H spectrum of compound **2b** (CD_2Cl_2, 400 MHz)

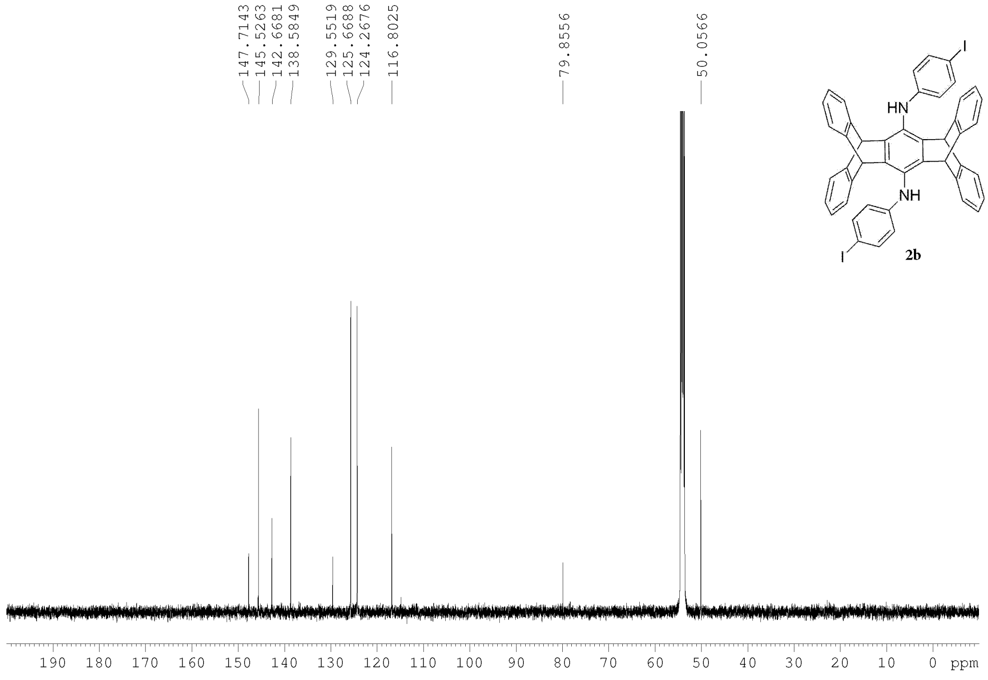

Figure S8. ^{13}C spectrum of compound **2b** (CD_2Cl_2, 126 MHz)

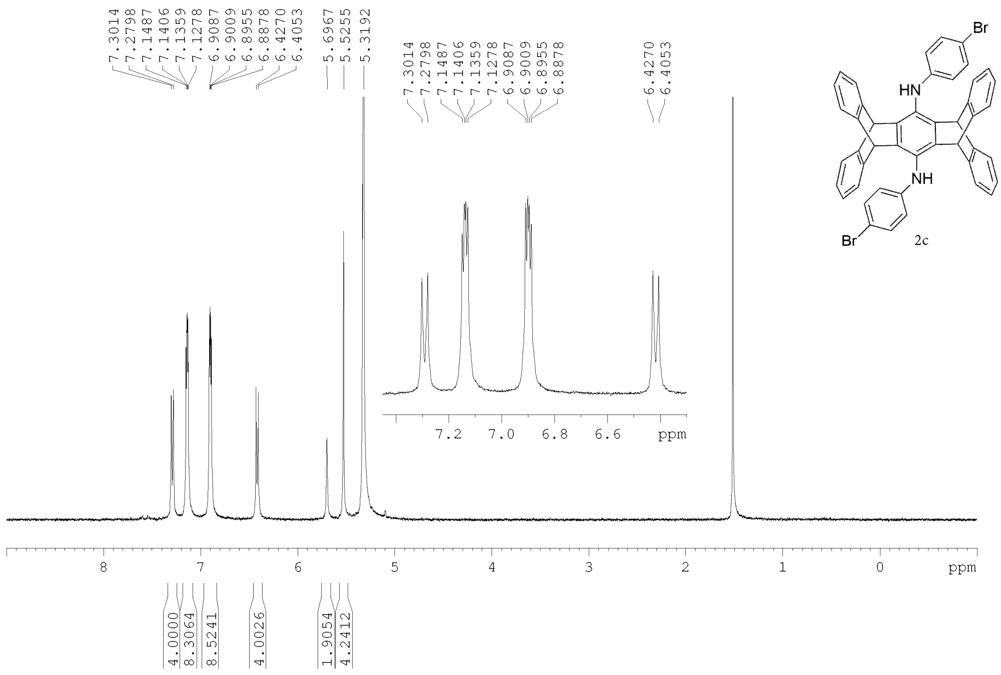

Figure S9. ^{1}H spectrum of compound **2c** (CD_2Cl_2, 400 MHz)

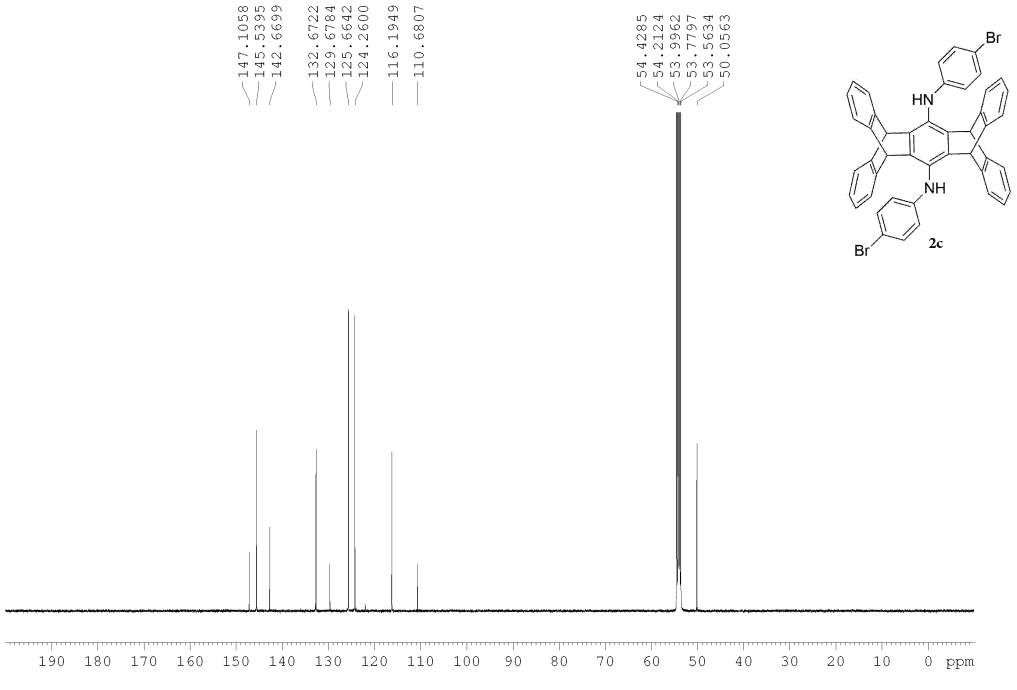

Figure S10. ^{13}C spectrum of compound **2c** (CD_2Cl_2, 126 MHz)

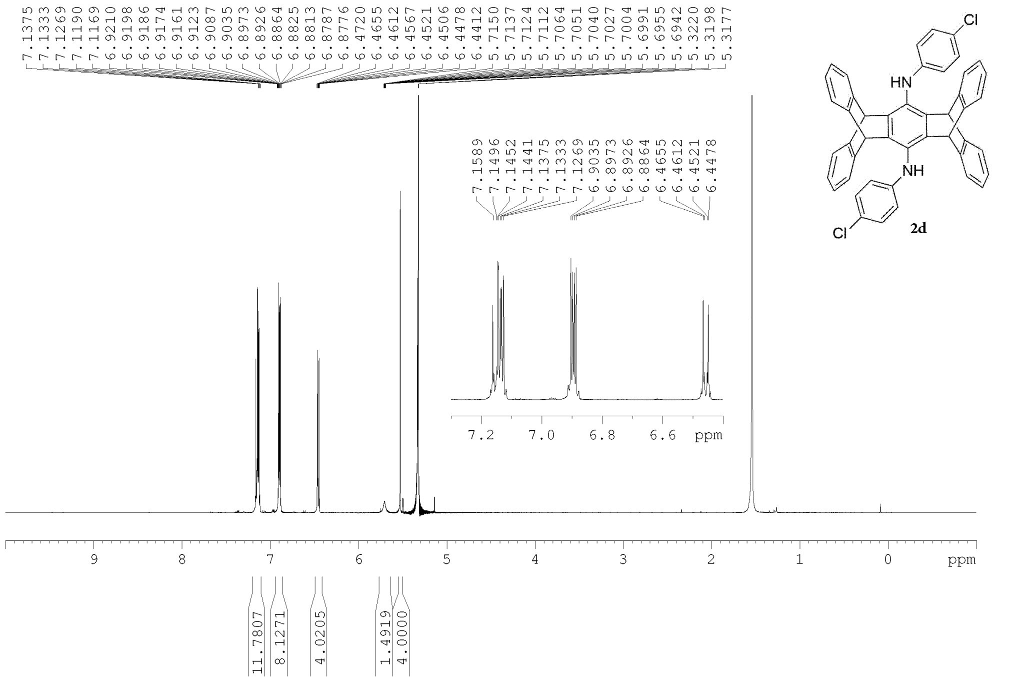

Figure S11. ^{1}H spectrum of compound **2d** (CD_2Cl_2, 500 MHz)

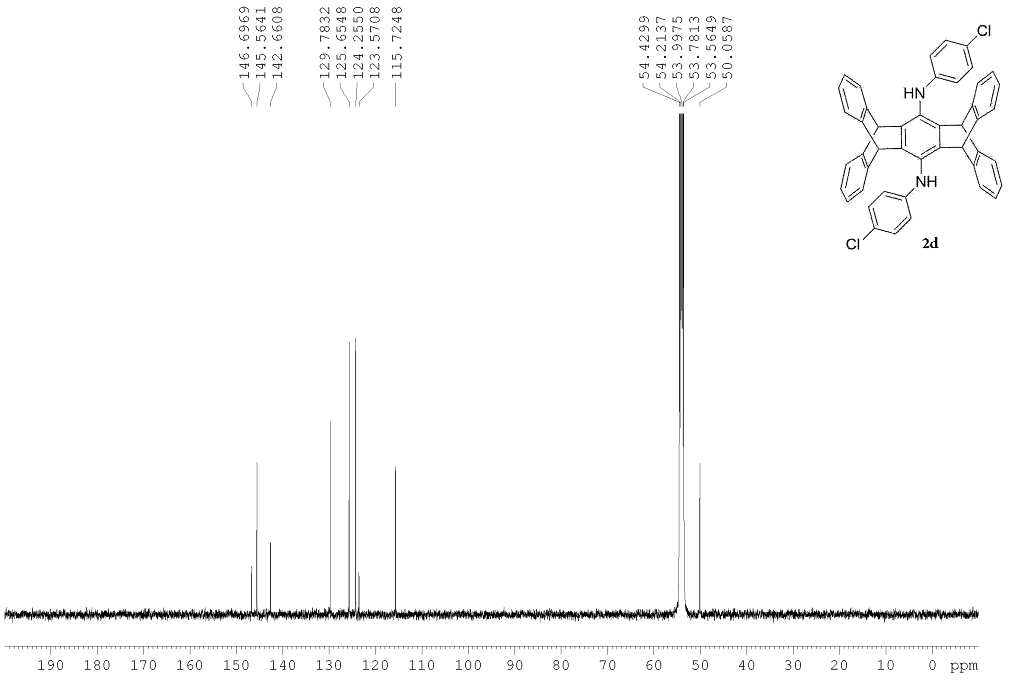

Figure S12. ^{13}C spectrum of compound **2d** (CD_2Cl_2, 126 MHz)

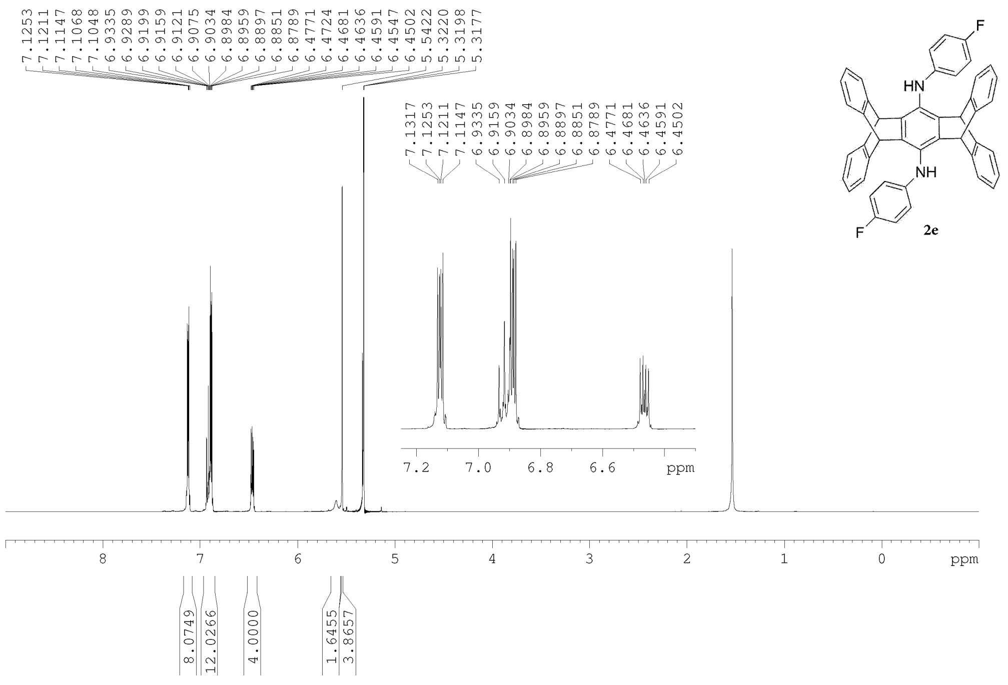

Figure S13. ^{1}H spectrum of compound **2e** (CD_2Cl_2, 500 MHz)

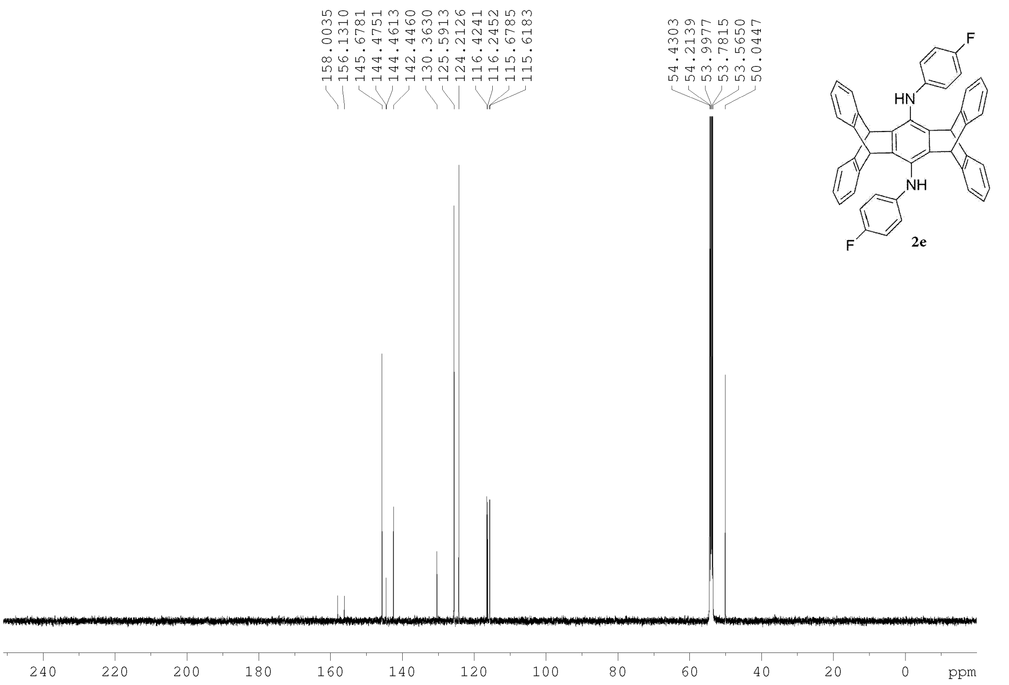

Figure S14. ^{13}C spectrum of compound **2e** (CD_2Cl_2, 126 MHz)

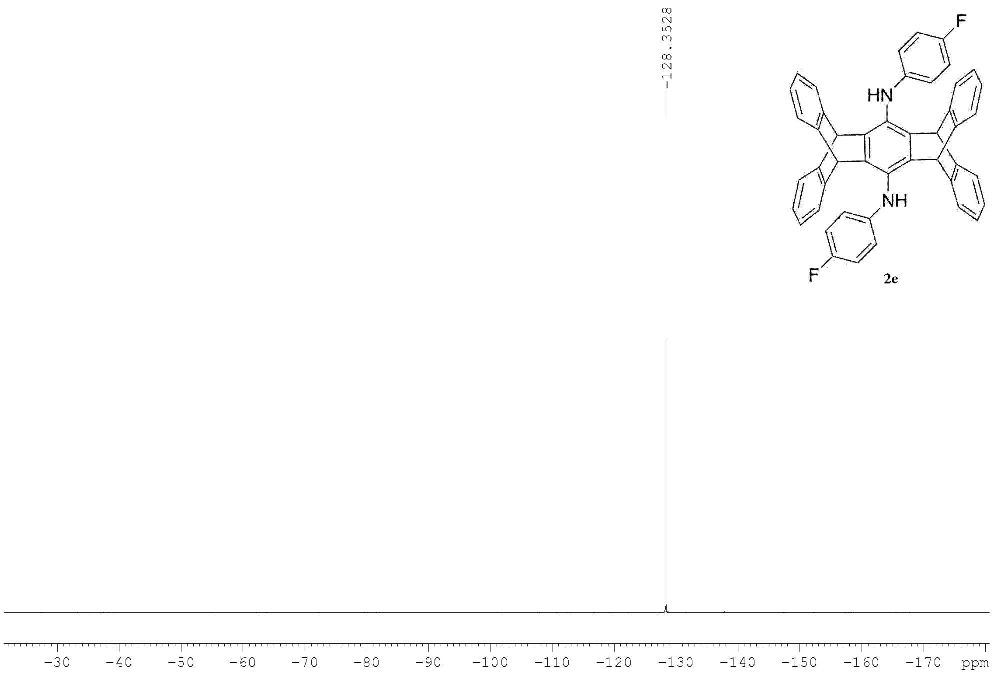

Figure S15. ^{19}F spectrum of compound **2e** (CD_2Cl_2, 471 MHz)

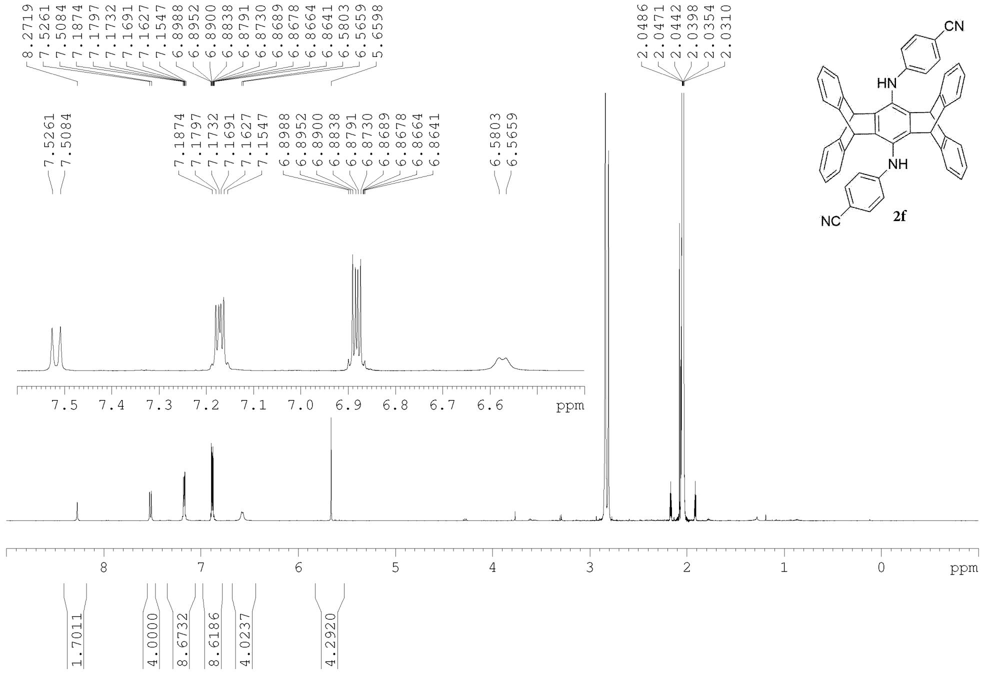

Figure S16. ^{1}H spectrum of compound **2f** (Acetone-d_6, 500 MHz)

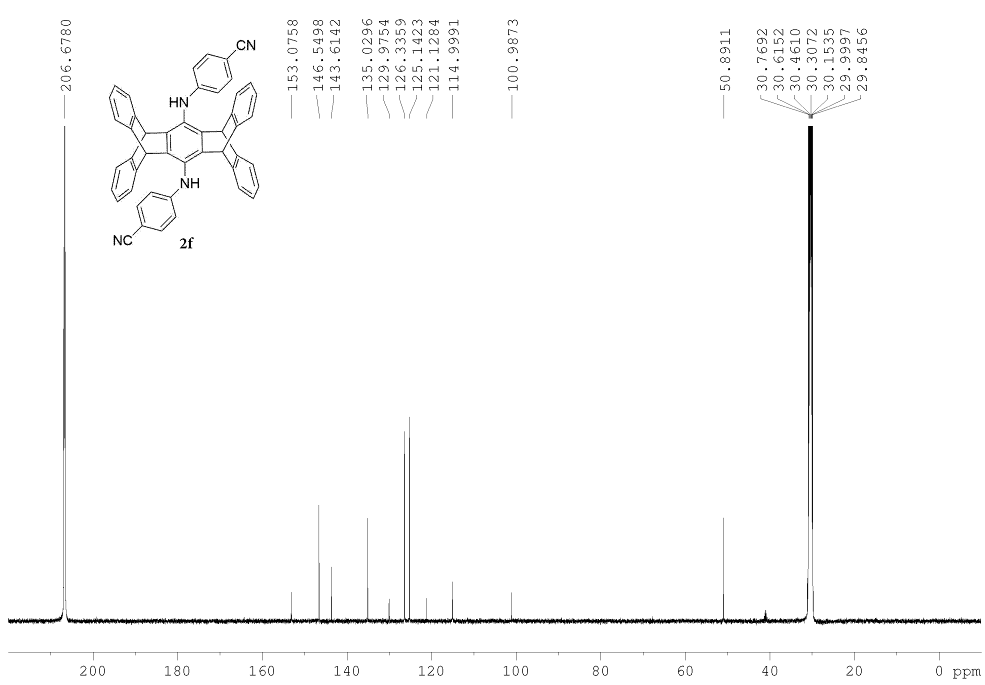

Figure S17. ^{13}C spectrum of compound **2f** (Acetone-d_6, 126 MHz)

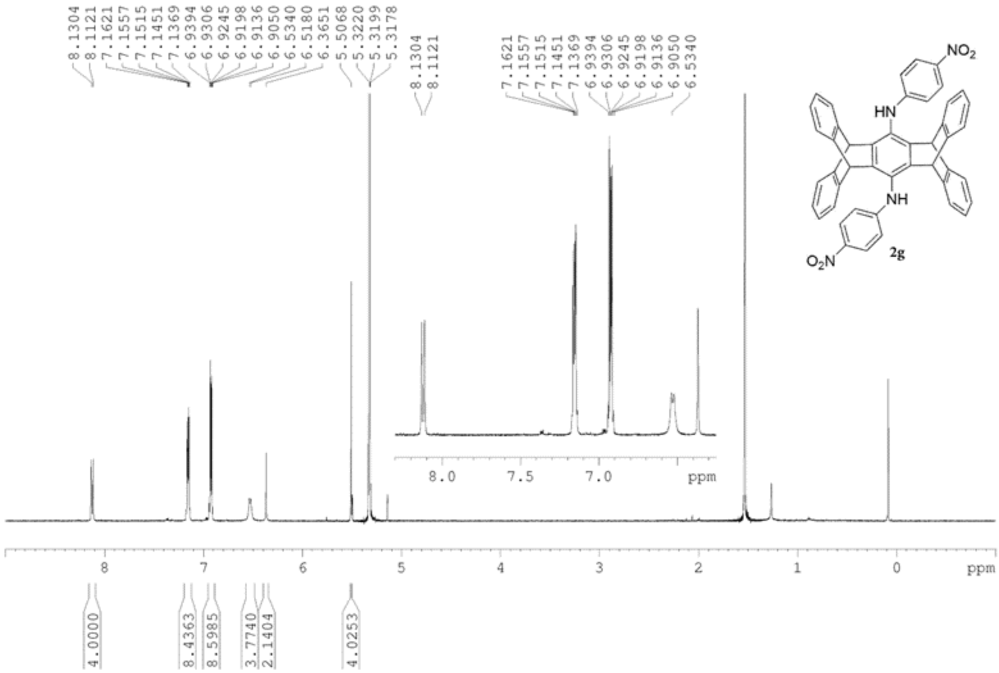

Figure S18. ^{1}H spectrum of compound **2g** (CD_2Cl_2, 500 MHz)

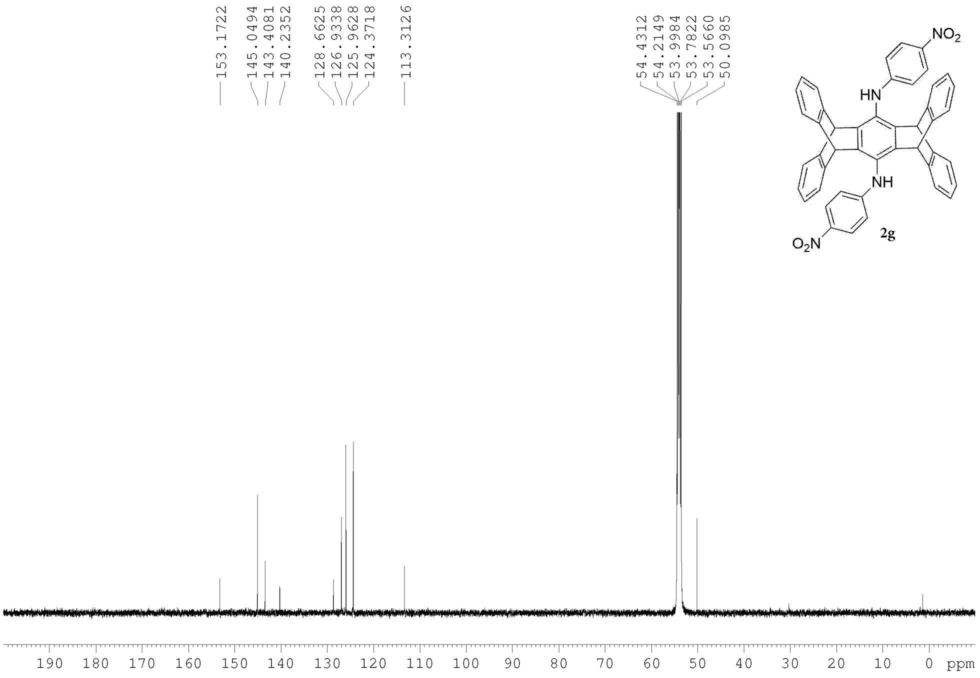

Figure S19. ^{13}C spectrum of compound **2g** (CD_2Cl_2, 126 MHz)

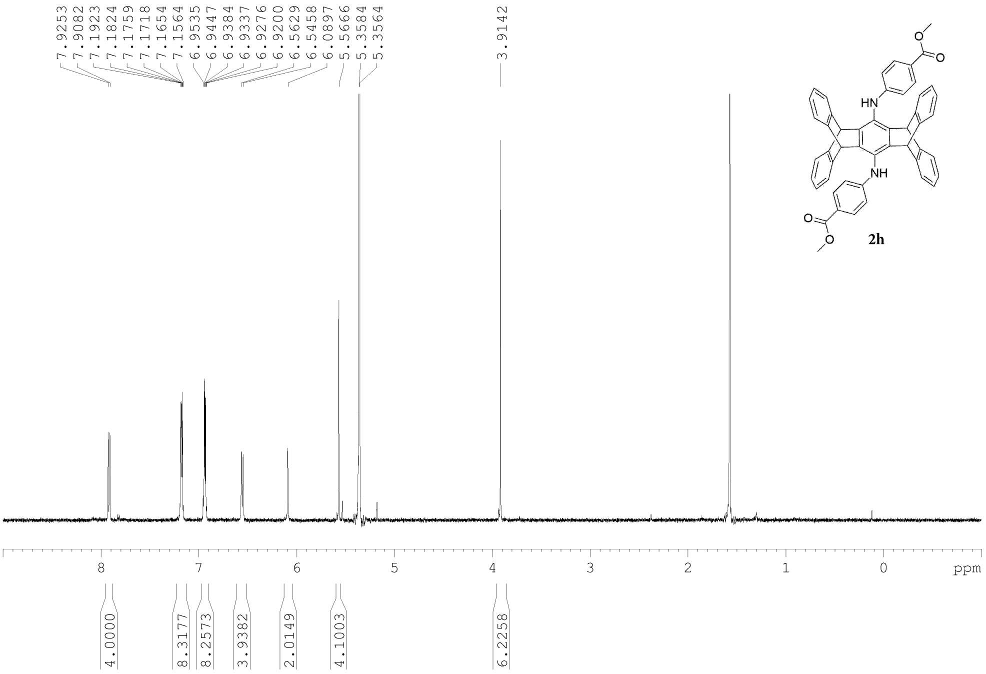

Figure S20. ^{1}H spectrum of compound **2h** (CD_2Cl_2, 500 MHz)

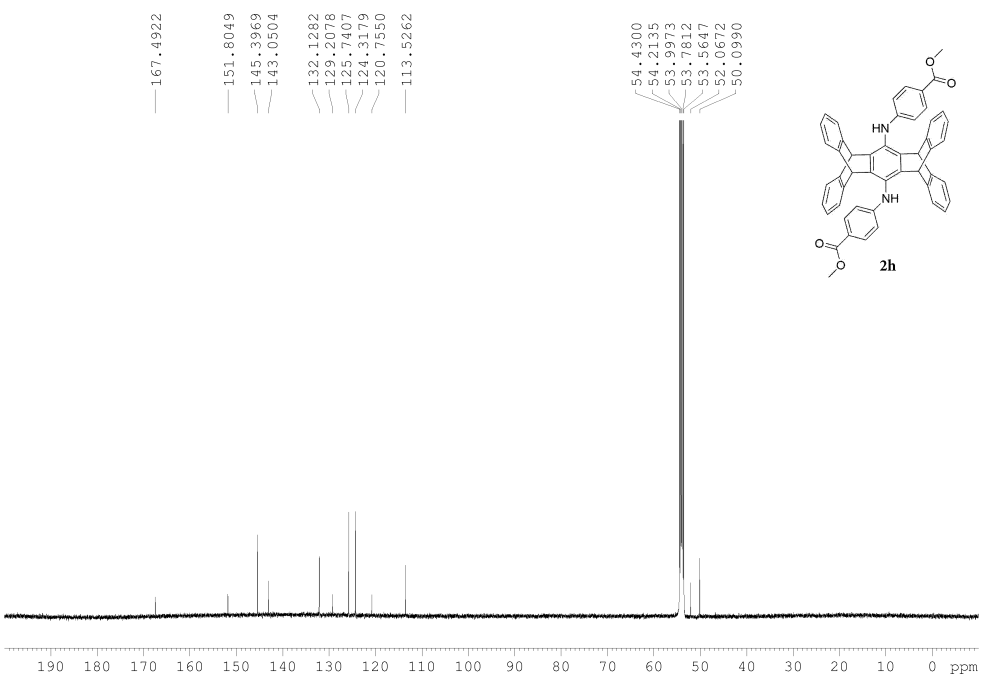

Figure S21. ^{13}C spectrum of compound **2h** (CD_2Cl_2, 126 MHz)

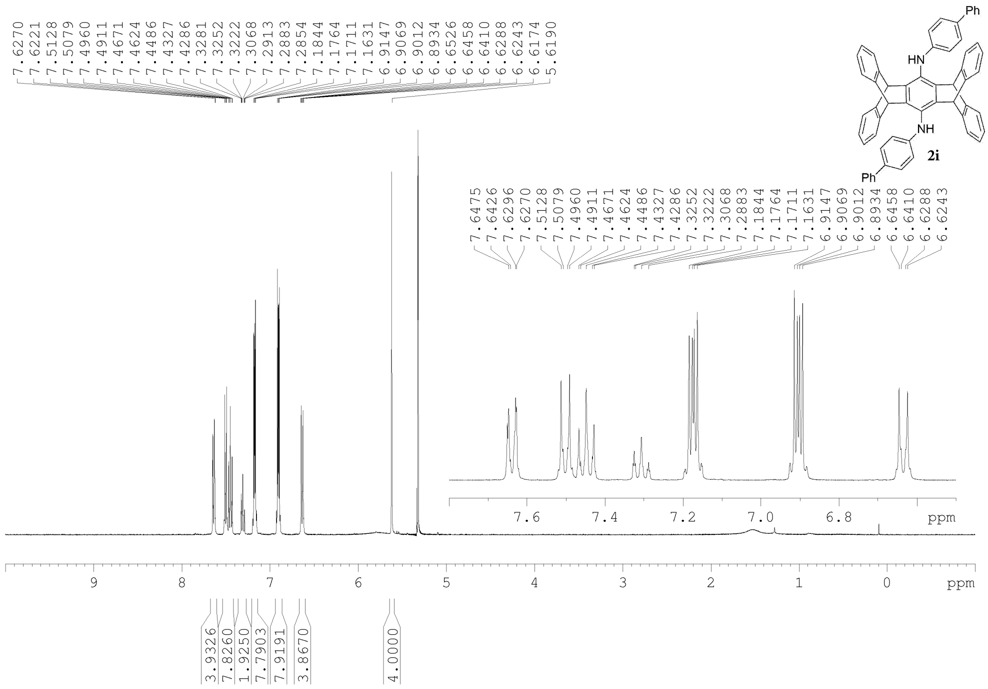

Figure S22. ^{1}H spectrum of compound **2i** (CD_2Cl_2, 400 MHz)

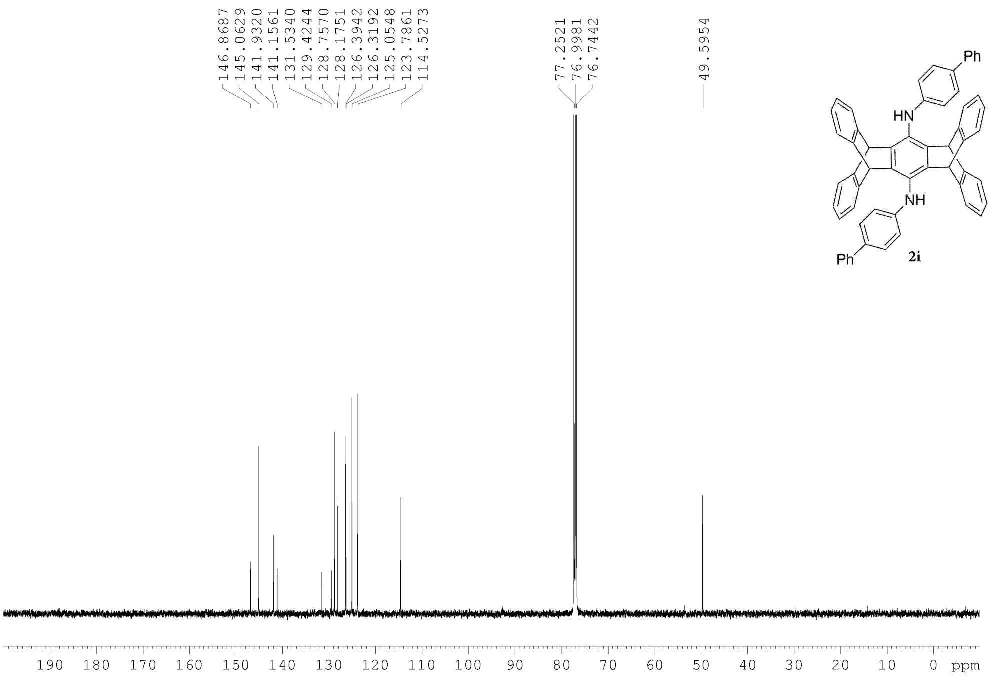

Figure S23. ^{13}C spectrum of compound **2i** ($CDCl_3$, 126 MHz)

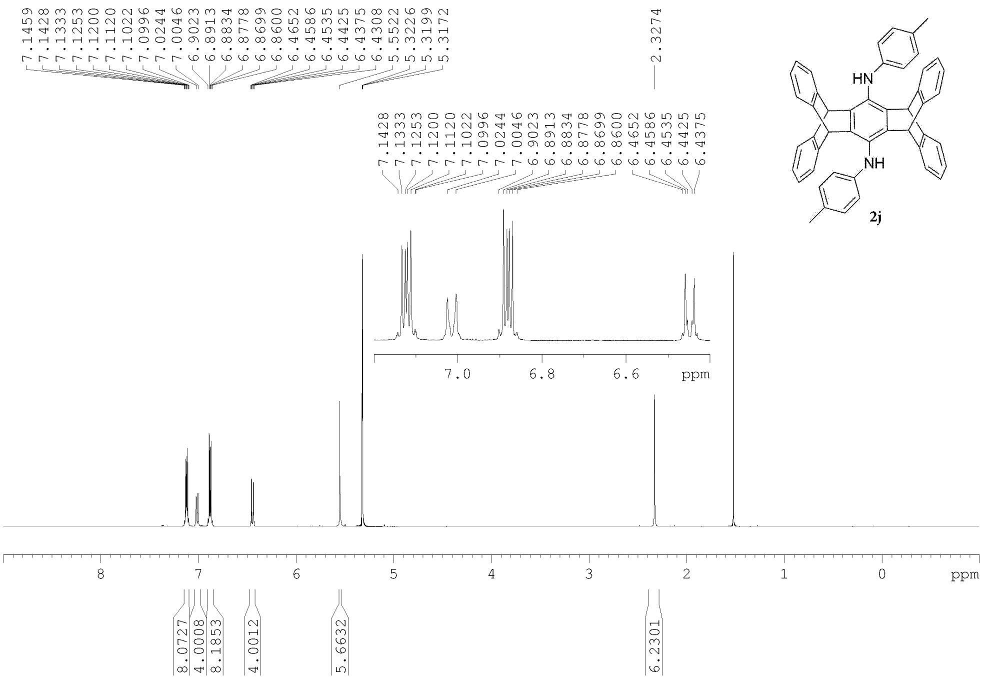

Figure S24. ^{1}H spectrum of compound **2j** (CD_2Cl_2, 400 MHz)

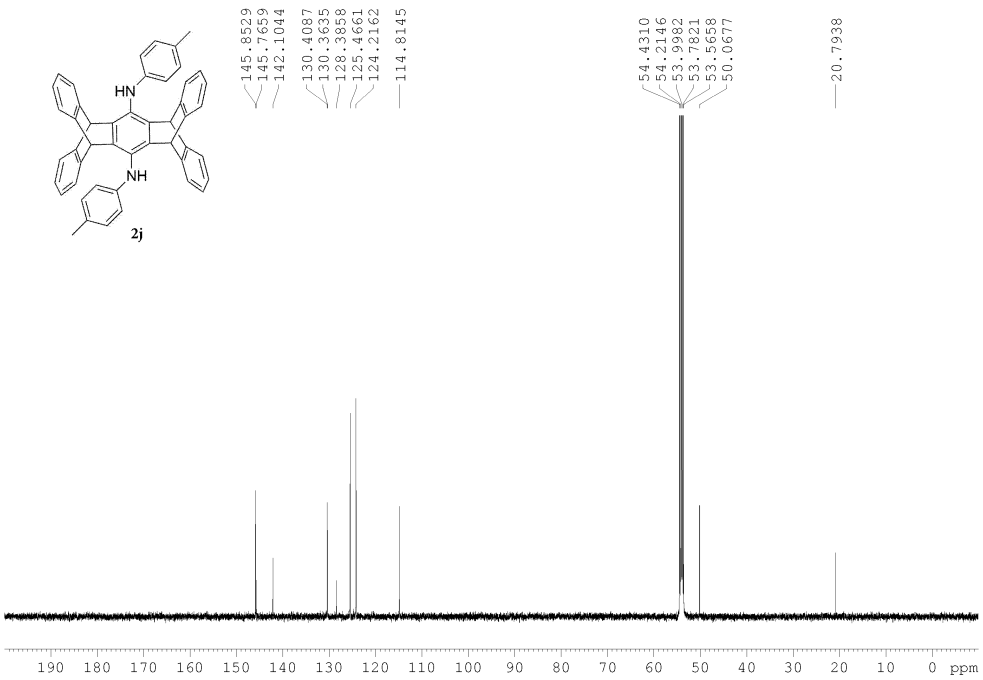

Figure S25. ^{13}C spectrum of compound **2j** (CD_2Cl_2, 126 MHz)

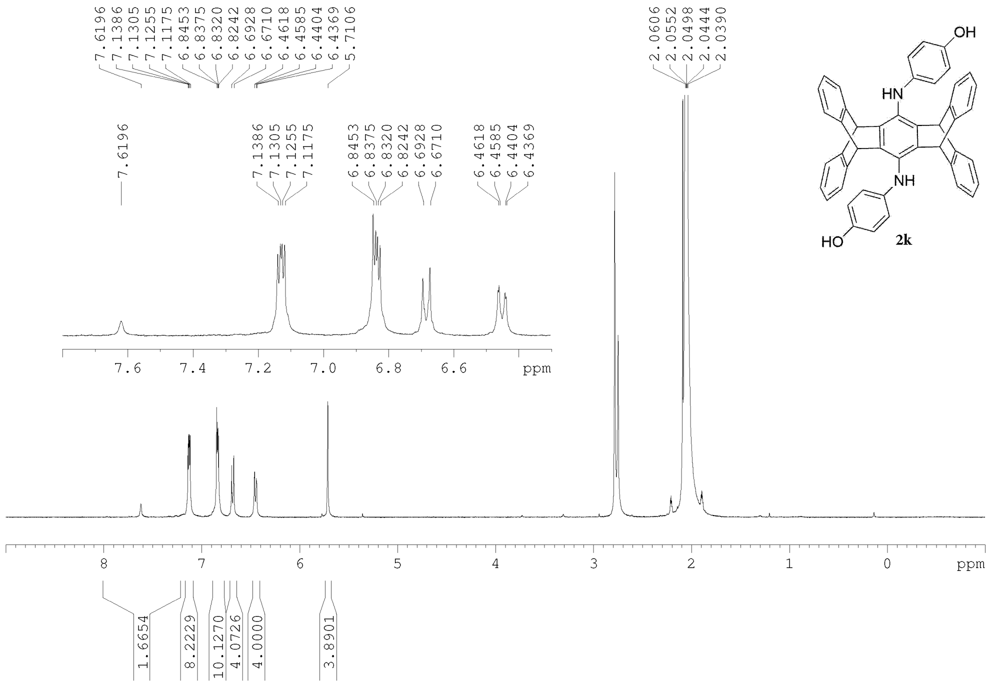

Figure S26. ^{1}H spectrum of compound **2k** (Acetone-d_6, 400 MHz)

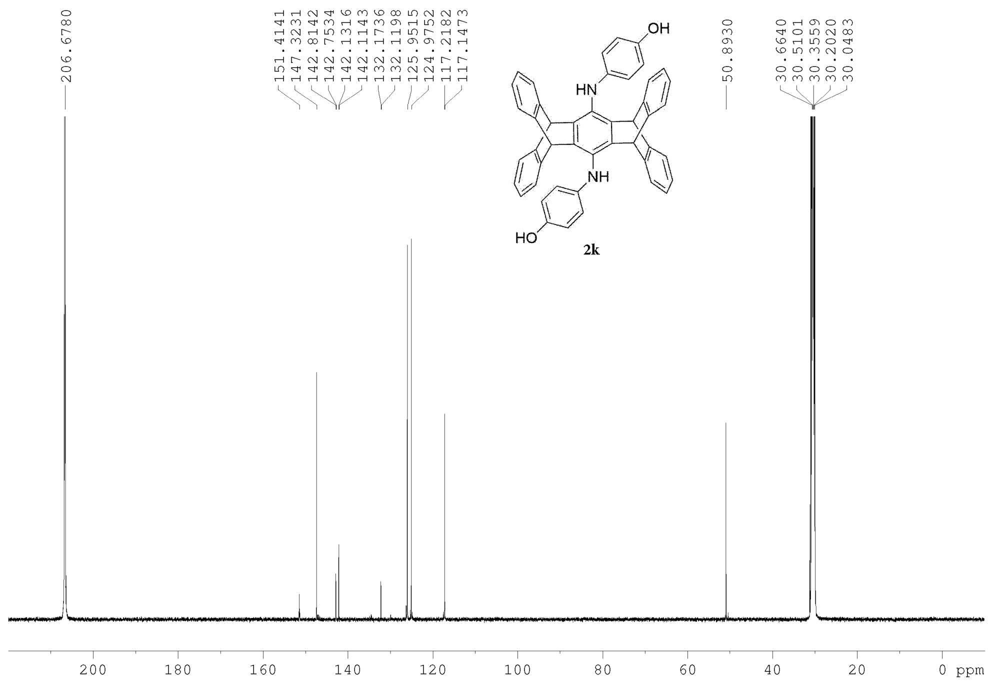

Figure S27. ^{13}C spectrum of compound **2k** (Acetone-d_6, 126MHz)

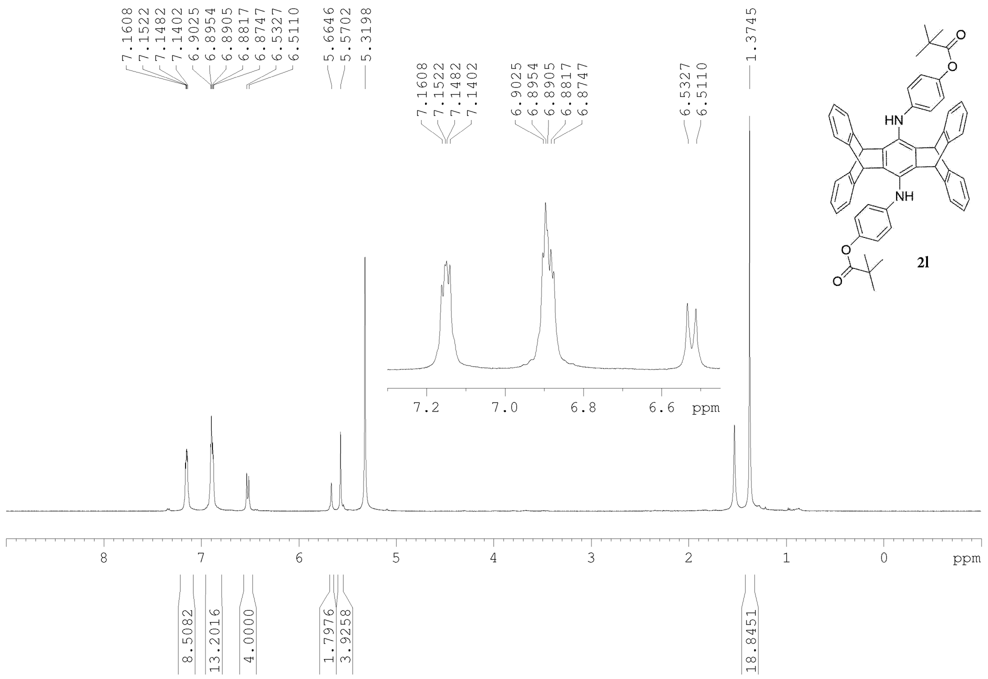

Figure S28. ^{1}H spectrum of compound **2l** (CD_2Cl_2, 400 MHz)

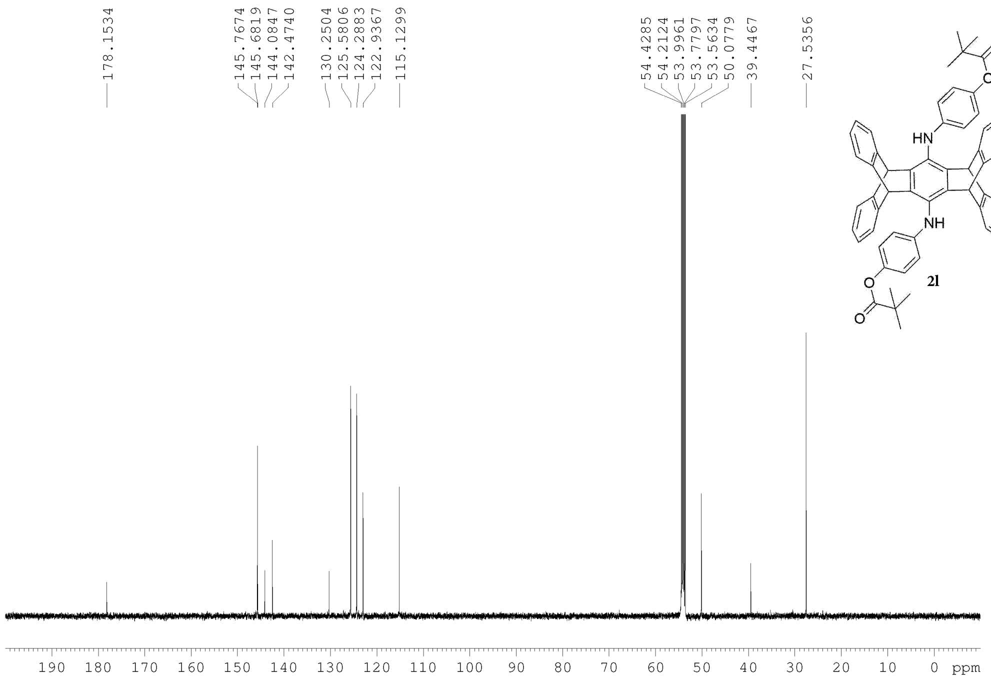

Figure S29. ^{13}C spectrum of compound **2l** (CD_2Cl_2, 126 MHz)

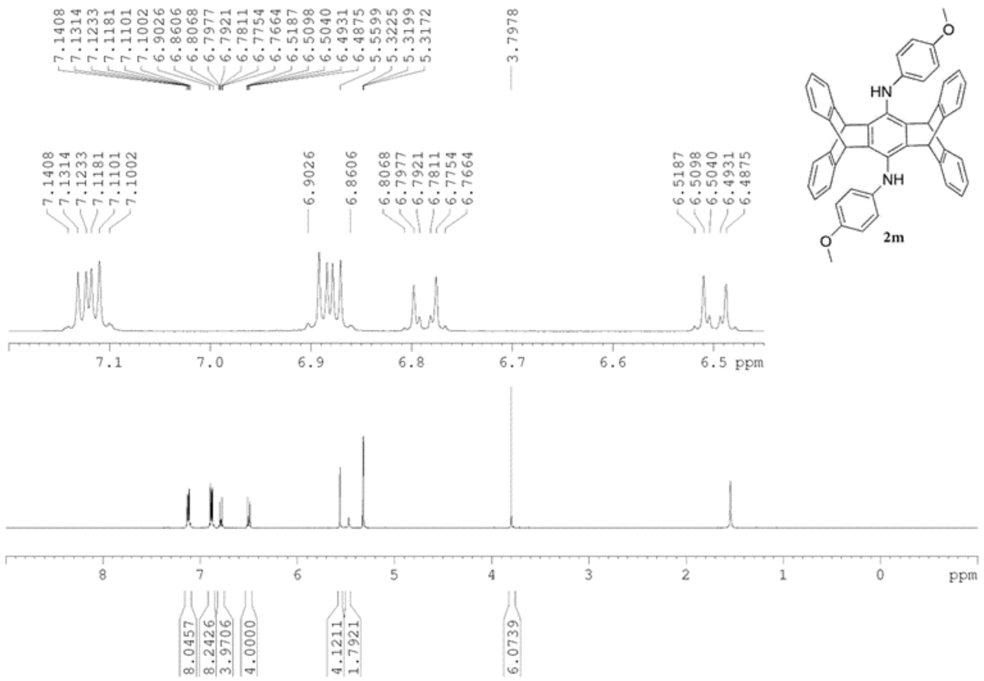

Figure S30. ^{1}H spectrum of compound **2m** (CD_2Cl_2, 400 MHz)

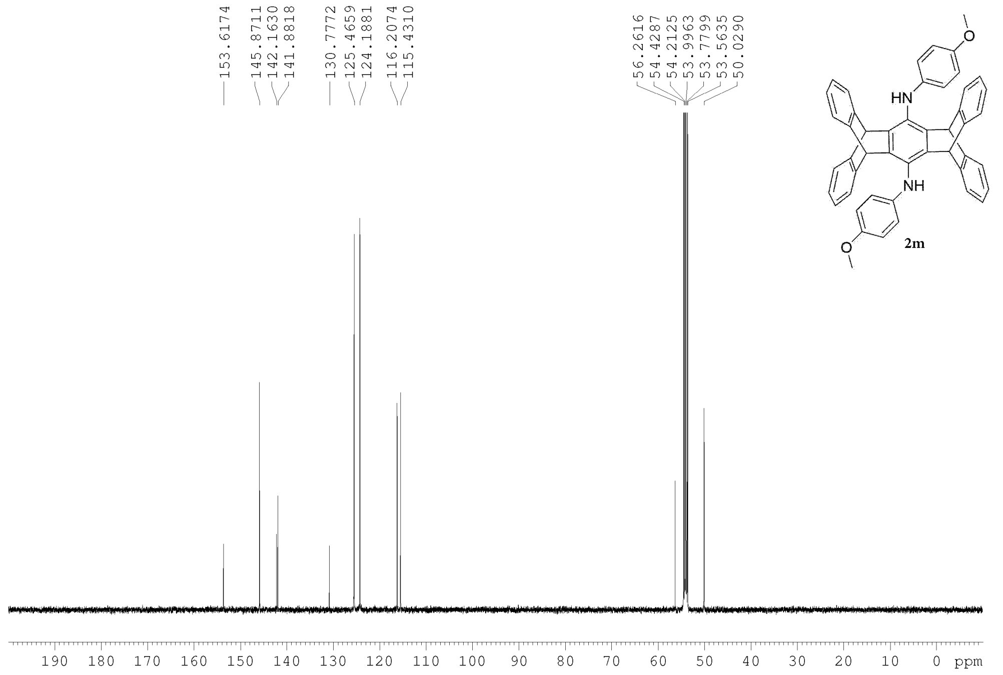

Figure S31. ^{13}C spectrum of compound **2m** (CD_2Cl_2, 126 MHz)

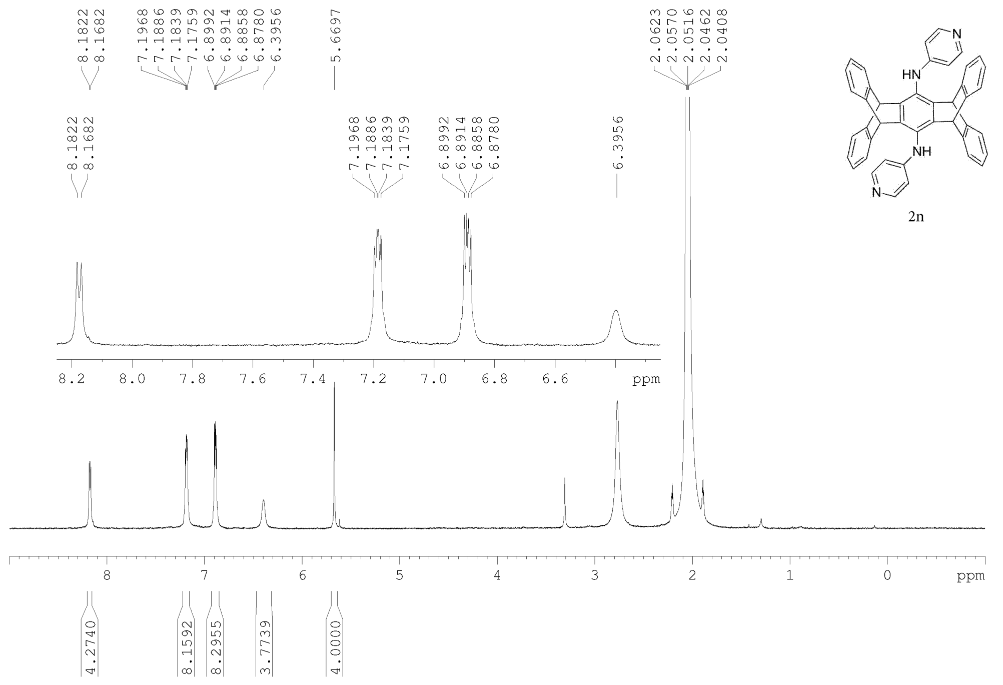

Figure S32. ^{1}H spectrum of compound **2n** (Acetone-d_6, 400 MHz)

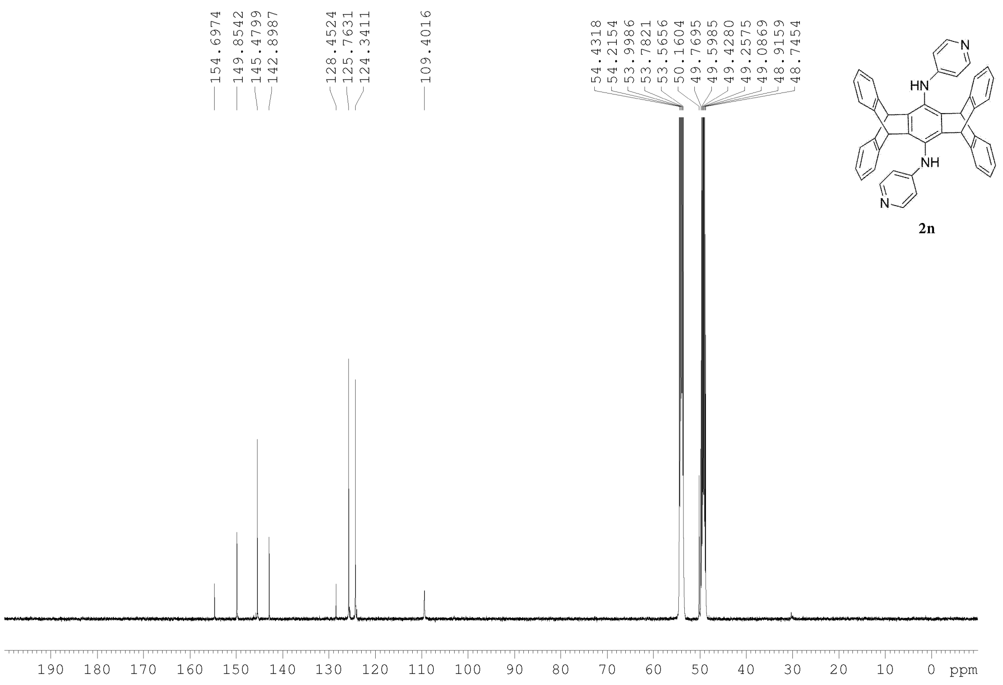

Figure S33. ^{13}C spectrum of compound **2n** (CD_2Cl_2/ CD_3OD = 5/1, 126 MHz)

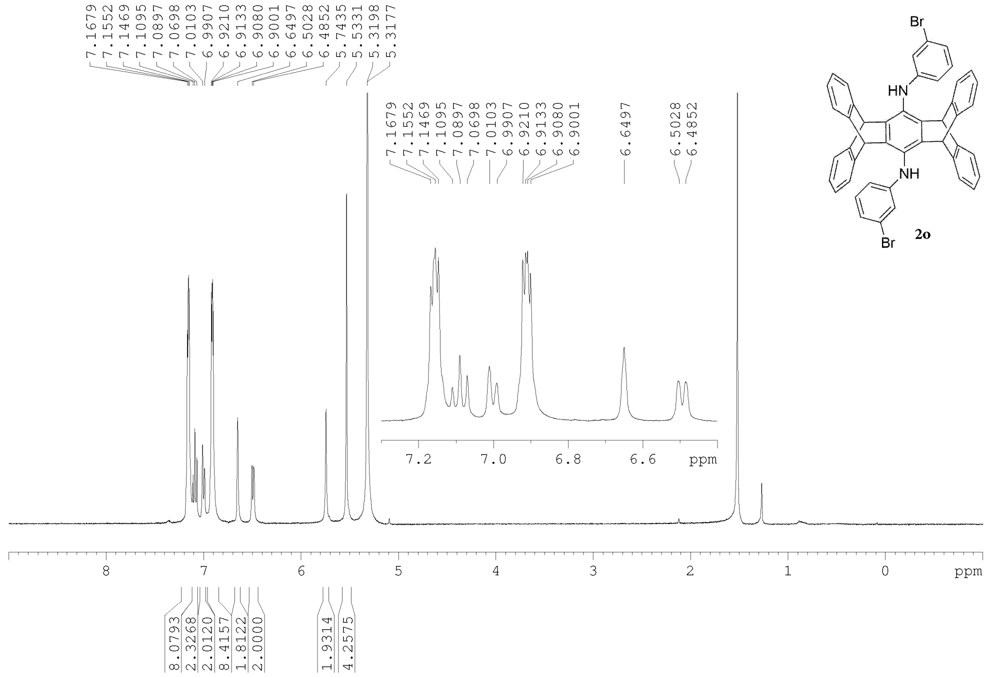

Figure S34. ^{1}H spectrum of compound **2o** (CD_2Cl_2, 400 MHz)

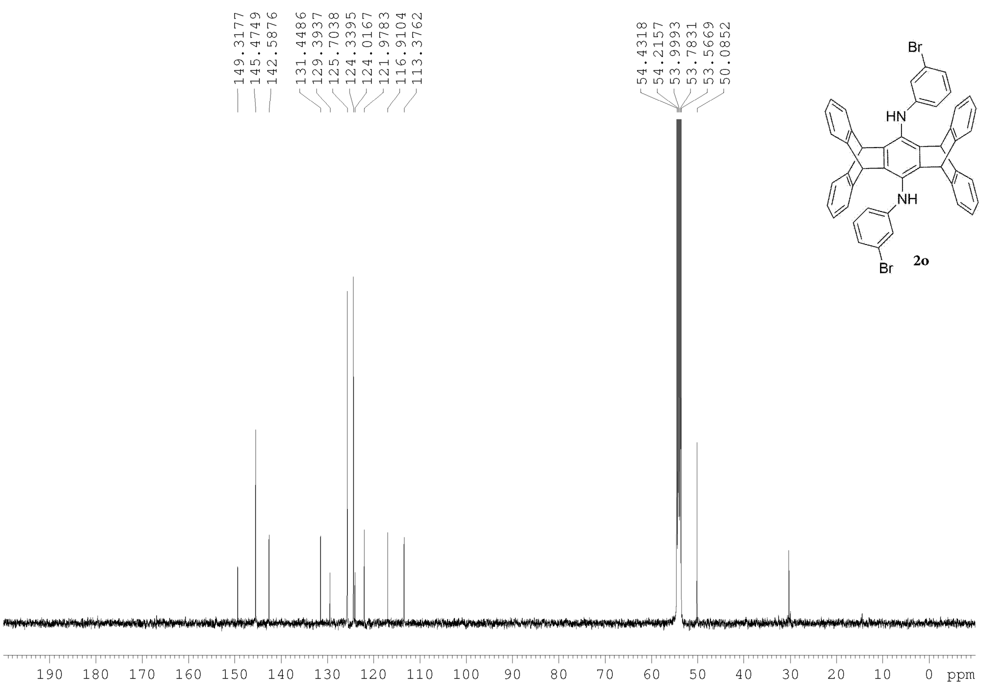

Figure S35. ^{13}C spectrum of compound **2o** (CD_2Cl_2, 126 MHz)

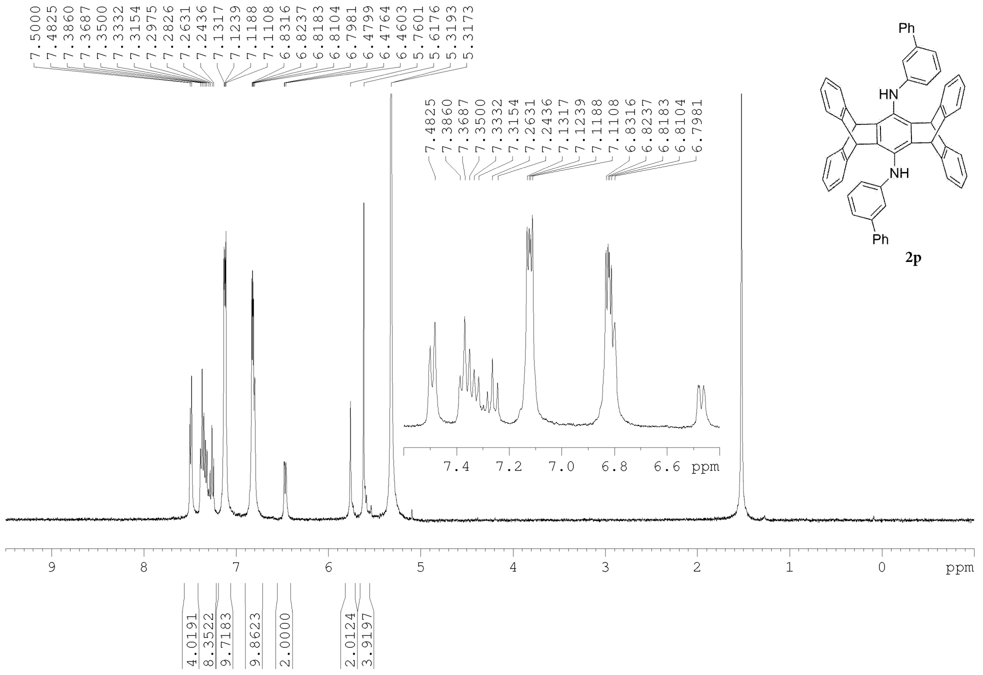

Figure S36. 1H spectrum of compound **2p** (CD_2Cl_2, 400 MHz)

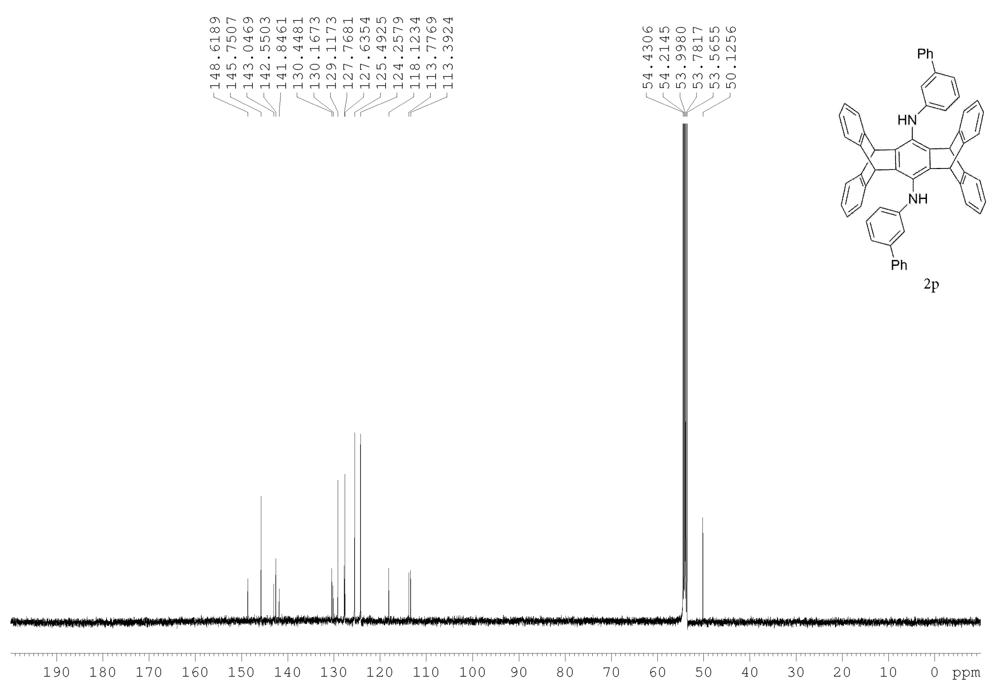

Figure S37. ^{13}C spectrum of compound **2p** (CD_2Cl_2, 126 MHz)

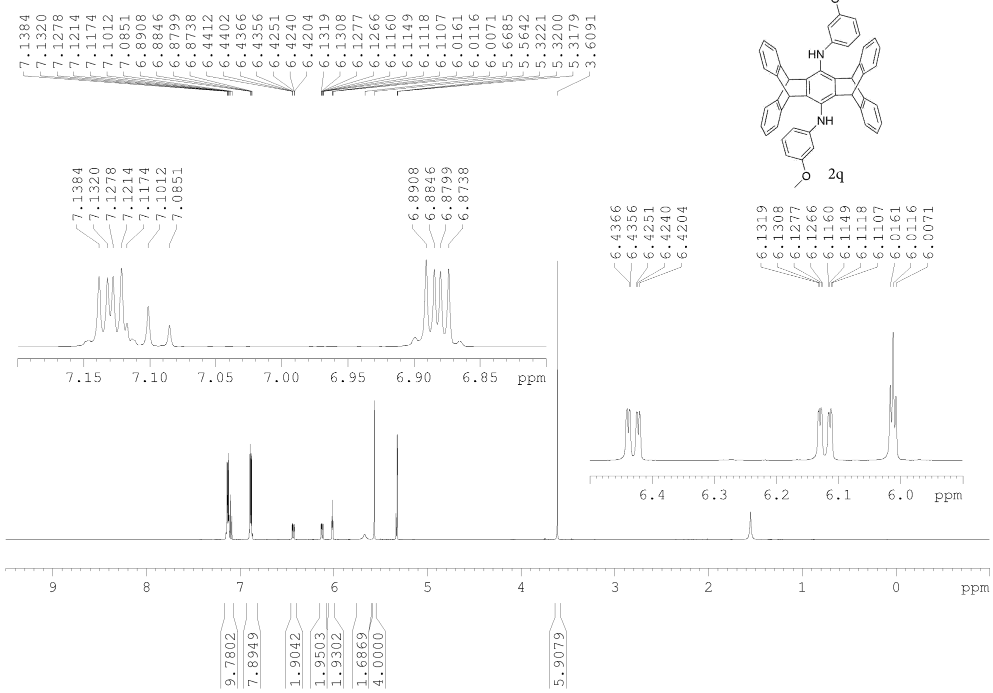

Figure S38. ^{1}H spectrum of compound **2q** (CD_2Cl_2, 500 MHz)

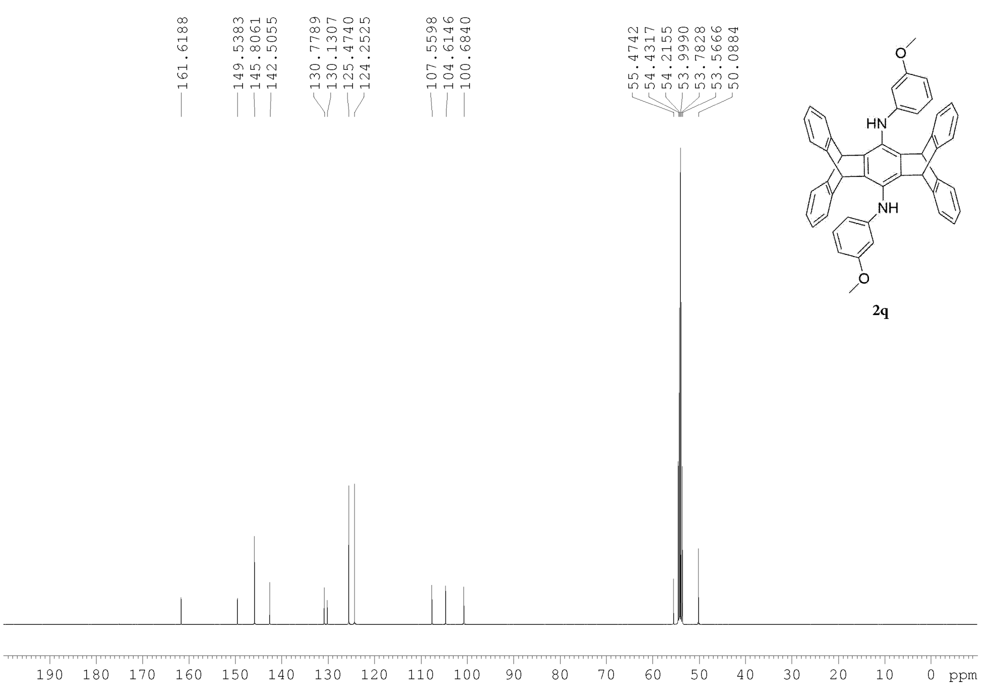

Figure S39. ^{13}C spectrum of compound **2q** (CD_2Cl_2, 126 MHz)

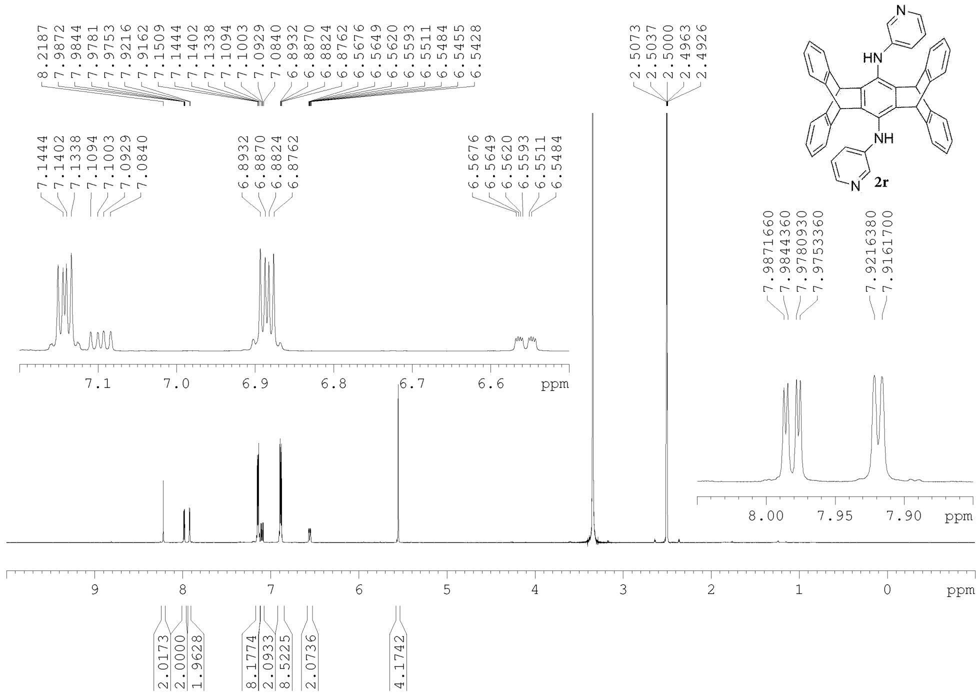

Figure S40. ^{1}H spectrum of compound **2r** (DMSO-d_6, 500 MHz)

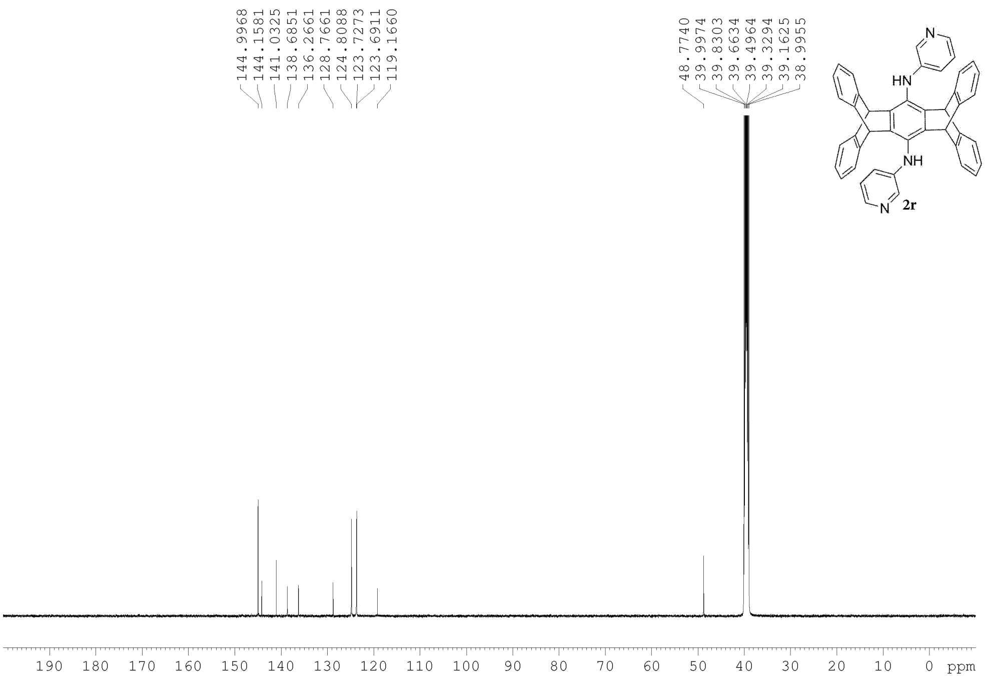

Figure S41. ^{13}C spectrum of compound **2r** (DMSO-d_6, 126 MHz)

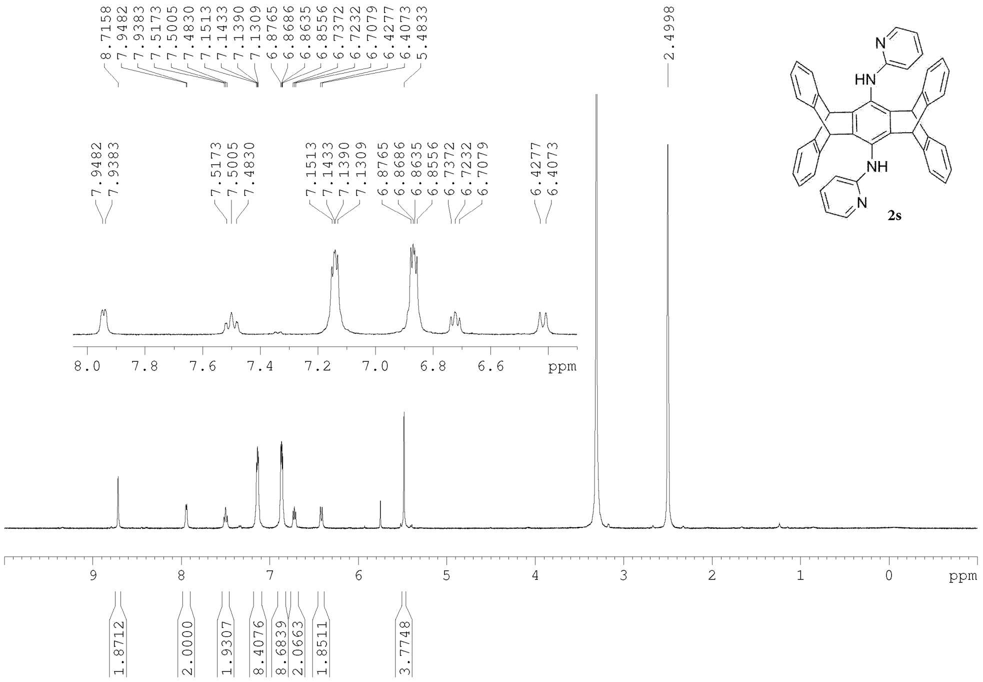

Figure S42. ^{1}H spectrum of compound **2s** (Acetone-d_6, 400 MHz)

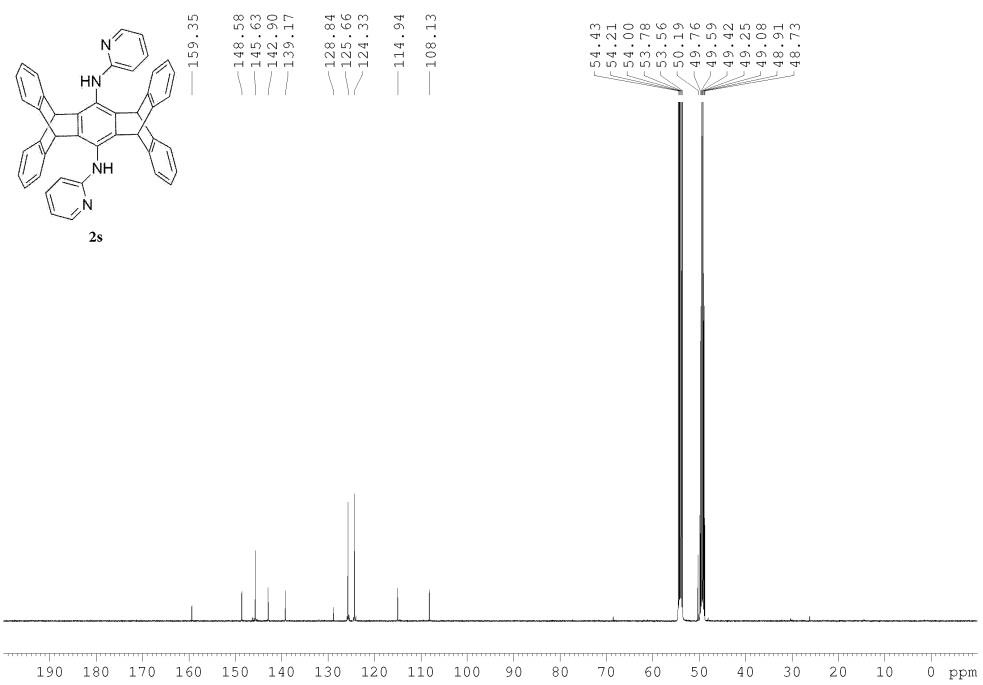

Figure S43. ^{13}C spectrum of compound **2s** (CD_2Cl_2/ CD_3OD = 5/1, 126 MHz)

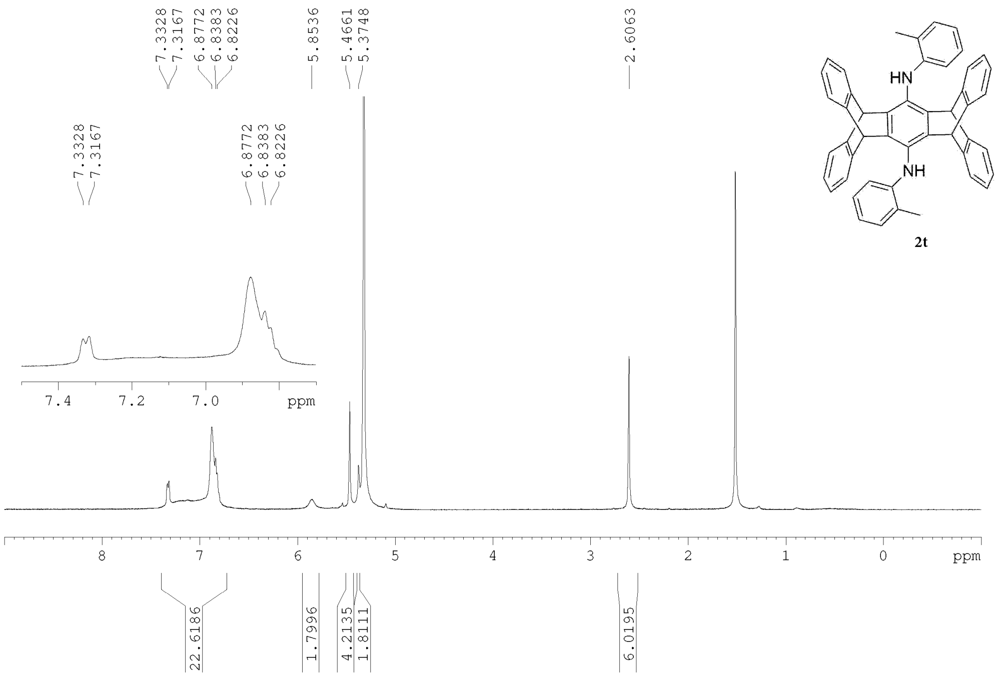

Figure S44. ^{1}H spectrum of compound **2t** (CD_2Cl_2, 400 MHz)

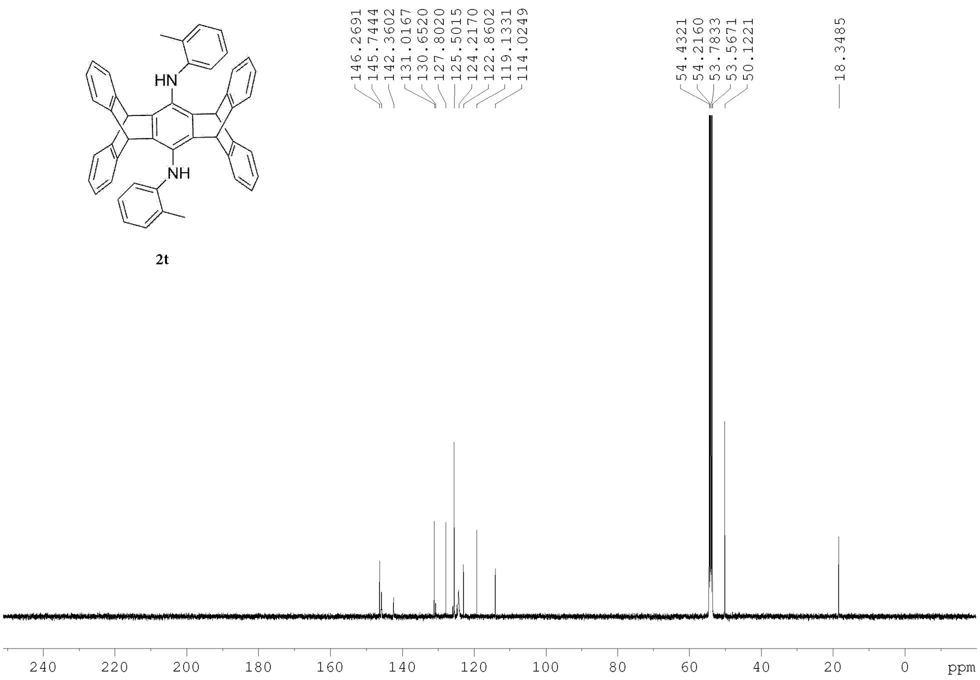

Figure S45. ^{13}C spectrum of compound **2t** (CD_2Cl_2, 126 MHz)

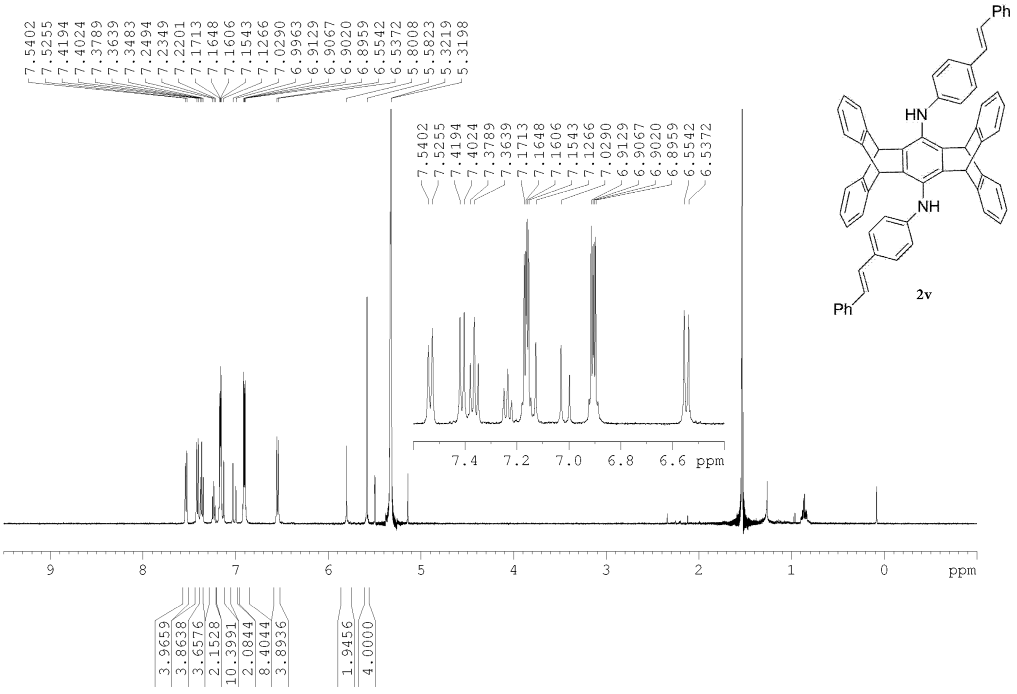

Figure S46. ^{1}H spectrum of compound **2v** (CD_2Cl_2, 500 MHz)

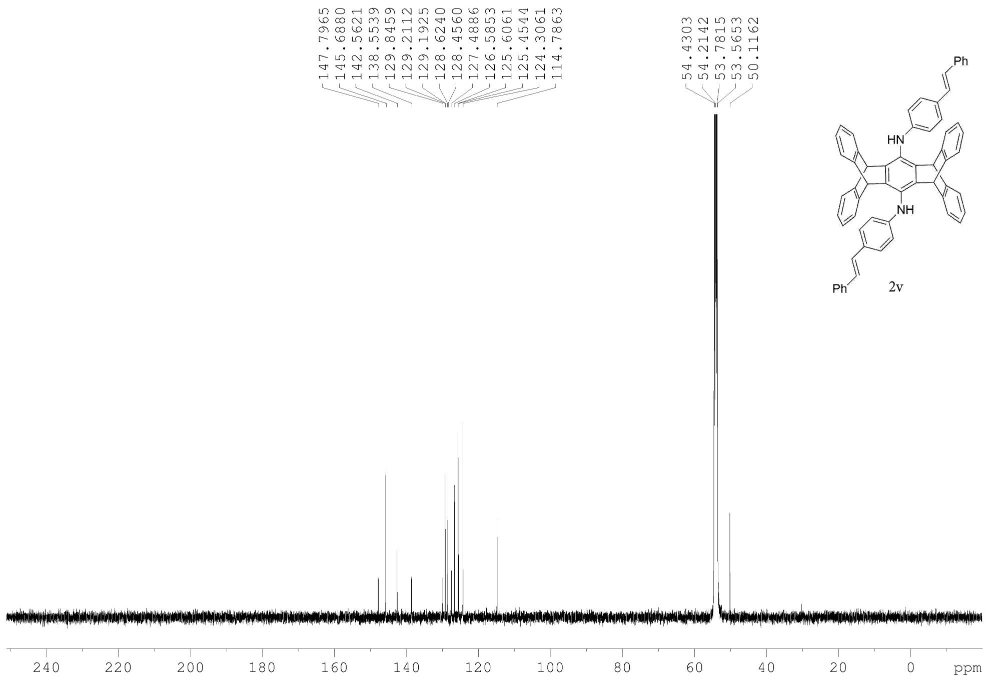

Figure S47. ^{13}C spectrum of compound **2v** (CD_2Cl_2, 126 MHz)

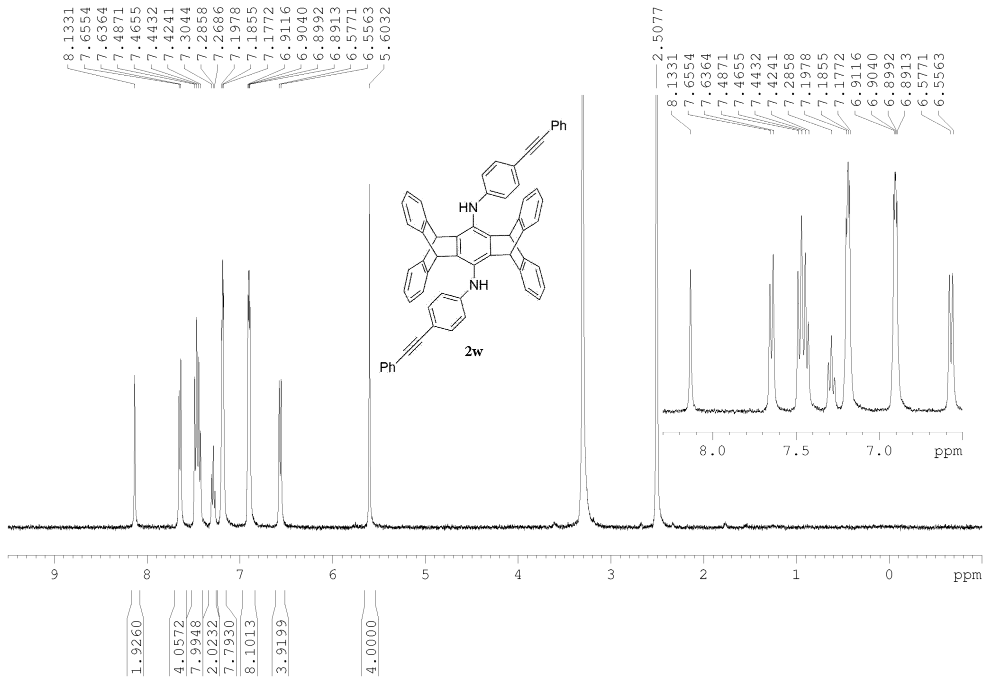

Figure S48. ^{1}H spectrum of compound **2w** (DMSO-d_6, 400 MHz)

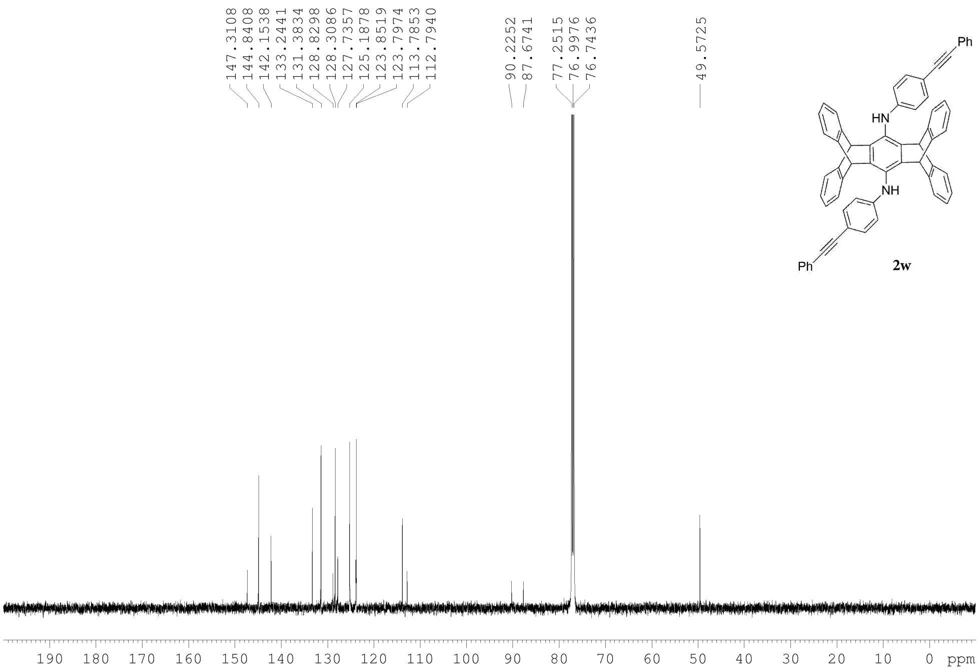

Figure S49. ^{13}C spectrum of compound **2w** ($CDCl_3$, 126 MHz)

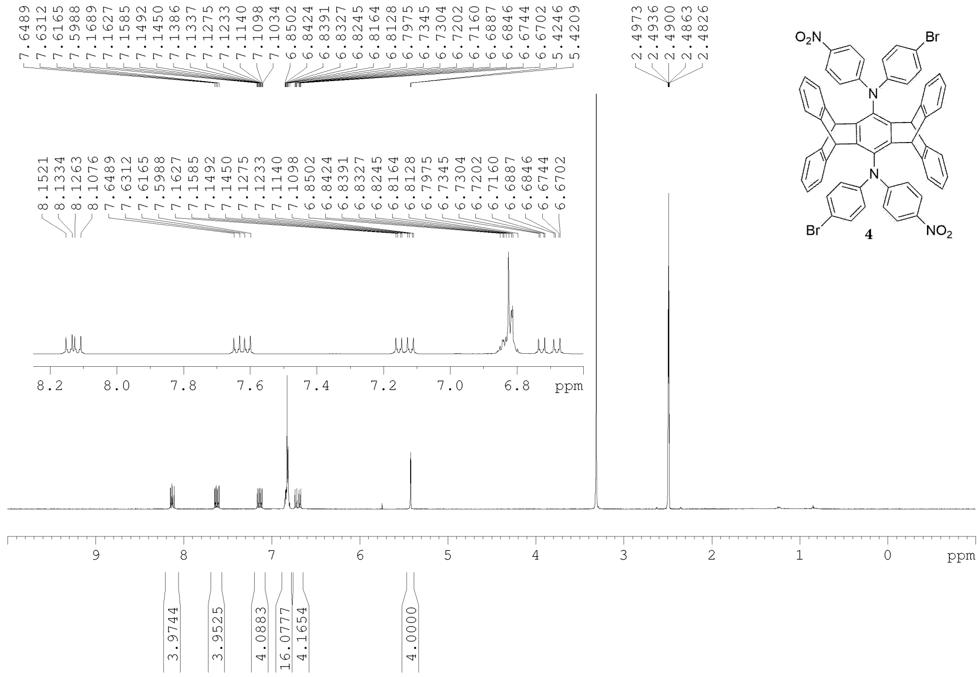

Figure S50. ^{1}H spectrum of compound **4** (DMSO-d_6, 500 MHz)

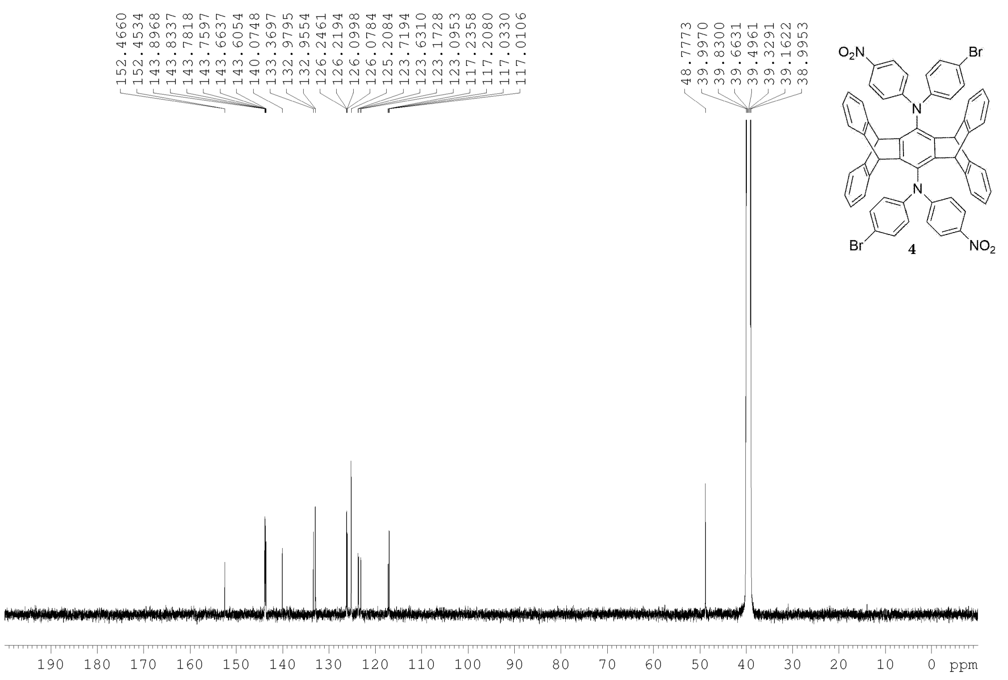

Figure S51. ^{13}C spectrum of compound **4** (DMSO-d_6, 126 MHz)

4. References

(S1) Zhu, X.-Z.; Chen, C.-F., Iptycene Quinones: Synthesis and Structure. *J. Org. Chem.* **2005,** *70*, 917-924.

(S2) Tan, W. S.; Lee, T.-Y.; Tseng, S.-F.; Hsu, Y.-F.; Ebina, M.; Taketsugu, T.; Huang, S.-J.; Yang, J.-S. "Additive-Dependent Iptycene Incorporation in Polyanilines: Insights into the Pentiptycene Clipping Effect and the Polymerization Mechanism" *J. Chin. Chem. Soc.* **2019**, *66*, 1141-1156.

(S3) Tan, W. S.; Lee, T.-Y.; Hsu, Y.-F.; Huang, S.-J.; Yang, J.-S., Iptycene substitution enhances the electrochemical activity and stability of polyanilines. *Chem. Commun.* **2018,** *54*, 5470-5473.

(S4) Yang, C.-H.; Prabhakar, C.; Huang, S.-L.; Lin, Y.-C.; Tan, W. S.; Misra, N. C.; Sun, W.-T.; Yang, J.-S., A Redox-Gated Slow-Fast-Stop Molecular Rotor. *Org. Lett.* **2011,** *13*, 5632-5635.

(S5) Kolla, H. S.; Surwade, S. P.; Zhang, X.; MacDiarmid, A. G.; Manohar S. K., Absolute Molecular Weight of Polyaniline. *J. Am. Chem. Soc.* **2005**, *127*, 16770-16771.
